# Epidemiological and clinical characteristics of long COVID-19 among Iranians: A community-based study in southern Iran

**DOI:** 10.1186/s12889-024-19543-1

**Published:** 2024-07-26

**Authors:** Mehrdad Askarian, Erfan Taherifard, Fatemeh Jazzabi, Zahra Shayan, Ojan Assadian, Gary Groot, Nahid Hatam, Ardalan Askarian, Seyed Mohammadebrahim Faghihi, Ehsan Taherifard

**Affiliations:** 1 Department of Community Medicine, Shiraz Medical School, Shiraz University of Medical Sciences, Shiraz, Iran; 2 MD‐MPH Department, Shiraz Medical School, Shiraz University of Medical Sciences, Shiraz, Iran; 3 Student Research Committee, Shiraz Medical School, Shiraz University of Medical Sciences, Shiraz, Iran; 4 Department of Biostatistics, Shiraz Medical School, Shiraz University of Medical Sciences, Shiraz, Iran; 5 Institute for Skin Integrity and Prevention, Regional Hospital Wiener Neustadt, Wiener Neustadt, Lower Austria Austria; 6 Department of Community Health and Epidemiology, College of Medicine, University of Saskatchewan, Saskatoon, Canada; 7 College of Arts & Science, University of Saskatchewan, Saskatoon, Canada; 8 Institute for Skin Integrity and Prevention, University of Huddersfield, Huddersfield, West Yorkshire UK

**Keywords:** Long COVID-19, Post-COVID-19 Condition, Post-Acute, Symptoms, SARS-CoV-2

## Abstract

**Background:**

The study aimed to evaluate the prevalence and pattern of long COVID-19 (LC) symptoms among individuals who had contracted COVID-19, to calculate the incidence of LC, and to provide insights into risk factors associated with developing LC in this population.

**Methods:**

This population-based cross-sectional survey was conducted in Fars province in 2023. Adult participants with a history of COVID-19 were recruited using a cluster random sampling method, alongside a control group with similar characteristics through the same methodology. Data were collected through in-person interviews using two researcher-developed data collection forms focused on demographic and clinical information.

**Results:**

A total of 2010 participants, comprising 1561 (77.7%) and 449 (22.3%) individuals with and without a previous history of COVID-19 were included. Among those with COVID-19 history, the prevalence of experiencing any symptoms was 93.7% (95% CI of 92.3%-94.8%) during the disease acute phase and 36.4% (95% CI of 34.0%-38.8%) after recovery. The incidence of symptoms specifically related to COVID-19, calculated by comparing the symptom rates between participants with and without a history of COVID-19, was found to be 13%. Factors such as older age, previous hospitalization for COVID-19, presence of cardiovascular disease, and use of steroids/chemotherapy were associated with LC symptoms.

**Conclusions:**

Our investigation sheds light on long-term aspects of COVID-19, demonstrating a significant prevalence of LC with diverse manifestations. It also underscores the importance of establishing standardized criteria and control groups in research on LC to address challenges related to heterogeneity and potential overestimation of symptoms.

**Supplementary Information:**

The online version contains supplementary material available at 10.1186/s12889-024-19543-1.

## Background

Severe acute respiratory syndrome coronavirus 2 (SARS‑CoV‑2) is a highly transmissible ribonucleic acid virus belonging to the Coronaviridae family. It emerged in the late 2019 and swiftly spread globally, precipitating nearly three years of the Coronavirus disease 2019 (COVID-19) pandemic [1, 2]. Over 750 million cases have been officially confirmed worldwide [3] since the onset of the pandemic. However, according to estimates from the SARS-CoV-2 evaluation model, the actual number of cases may soar into billions due to a large proportion of unconfirmed individuals [4]. Since the initial outbreak, the world has experienced multiple waves of COVID-19, driven by emerging variants of the virus. Each wave brought distinct challenges, characterized by varying rates of transmission, severity, and impacts on healthcare systems [5]. In Iran, as well, the pandemic's trajectory mirrored global trends, with distinct waves marked by unique epidemiological and clinical characteristics [6]. A large cohort study analyzing the demographic and clinical characteristics of 24,287 hospitalized patients across six waves of COVID-19 in Northern Iran revealed that the highest in-hospital mortality rates occurred during earlier waves of the pandemic. The fifth wave, notable for involving younger patients, had the lowest mortality rate and ICU admissions [7]. Based on the latest official statistics, over seven million cases of COVID-19 were reported in Iran, with 146,811 fatalities [8].

The clinical spectrum of acute infection with SARS-CoV-2 ranges from asymptomatic forms to symptomatic forms, with most patients experiencing fever, cough, dyspnea, myalgia, and headache, alongside severe complications, including acute respiratory distress syndrome, sepsis, and multi-organ failure in critical cases [5]. Manifestations in this stage derive primarily from viral replication, direct virus-mediated tissue damage, and subsequently from inflammatory cell recruitment [5]. Beyond the well-documented acute phase of the infection, a broad spectrum of persistent or newly emerging symptoms following recovery from the initial infection has been observed, collectively termed as long COVID-19 (LC). This phenomenon presents a challenge to our comprehension of the virus's long-term impact on individuals' health [9]. Proposed underlying pathogenic mechanisms for LC include viral persistence and dormancy, autoimmunity with an uncoordinated immune response, systemic and tissue-specific inflammation, and microvascular dysfunction [10–14]. It presents with a complex array of symptoms including fatigue, cognitive and psychiatric difficulties, and shortness of breath, myalgia, and other persistent manifestations, totaling over two hundred reported symptoms that can impair daily functioning. The trajectory of LC symptoms over time is varied and unpredictable, often persisting for months [9, 15].

This phenomenon has prompted extensive research and clinical attention as the medical community endeavors to comprehend, manage, and provide support for individuals experiencing lingering effects long after the initial infection. However, due to the heterogeneity of study protocols, including variations in included symptoms and targeted patient populations, estimating the prevalence of LC proves challenging. Given the global prevalence of SARS-CoV-2 and the significant percentage of individuals experiencing these long-term manifestations, the burden of LC is substantial. There is a significant paucity of studies focusing on persistent symptoms and associated risk factors of LC in Iran. Most available studies suffer from limitations such as small sample sizes and the absence of comparator groups [16, 17], which restricts the ability to draw comprehensive conclusions about the true prevalence and impact of LC. This study will utilize a cross-sectional design with a control group to provide a comprehensive understanding of the epidemiological and clinical characteristics of LC in the Iranian population. The objectives include delineating both new and persistent symptoms among Iranian patients with SARS-CoV-2 in the subacute and chronic phases, estimating the incidence of LC in this demographic, identifying associated risk factors, and assessing the burden and impact of LC on daily functioning.

## Methods

We conducted a population-based cross-sectional survey to assess prevalence and incidence of symptoms in individuals with a history of SARS‑CoV‑2 infection during the acute phase and subsequently as part of LC, and to determine associated factors, while comparing with matched control group. The survey was conducted in Fars province from June to August 2023. The Institutional Review Board of Shiraz University of Medical Sciences approved the protocol (IR.SUMS.MED.REC.1402.055). The study adhered to the principles of the Declaration of Helsinki. Participants voluntarily joined the survey, and informed consent was obtained from all of them, with the right to withdraw consent at any time.

### Study participants

Participants eligible for inclusion were: (1) individuals aged 18 years or older, (2) a history of SARS‑CoV‑2 infection with a minimum one-month interval prior to the interview, (3) individuals residing in Fars province at the time of interviewing, and (4) individuals who had provided oral-informed consent.

A positive history of SARS‑CoV‑2 infection was determined in any of the following conditions: (1) confirmation through a positive test (PCR, rapid test), (2) diagnosis based on medical advice from physicians through medical history, examinations or chest CT scan, and (3) endorsement by strong personal suspicion, such as close contact with a confirmed SARS‑COV‑2 case. Individuals unable to participate in direct interviews due to physical or mental illness, or those unavailable during the data collection period, were excluded.

### Sample size determination

Using the ($\frac{{Z}^{2}\times P\times (1-P)}{{d}^{2}}$) formula, with a confidence level of 95%, an expected LC prevalence of 30% [18], and a margin of error of 5%, the required sample size for our patient group was estimated to be 323. For the control group size calculation, considering an expected general symptom prevalence of 15% in controls [19], an expected LC prevalence of 30% in cases, a confidence level of 95%, and a power of 80%, the optimal sample size was determined to be approximately 118, using the ($\frac{{(Z}_{\alpha /2}+ {Z}_{\beta }) \times ({P}_{1}\left(1-{P}_{1}\right)+ {P}_{2}\left(1-{P}_{2}\right))}{{({P}_{1}-{P}_{2})}^{2}}$) formula [20, 21]. Thus, the final sample size for the study, including both groups, was calculated to be at least 441 individuals.

### Sampling method

**Fig. 1:**
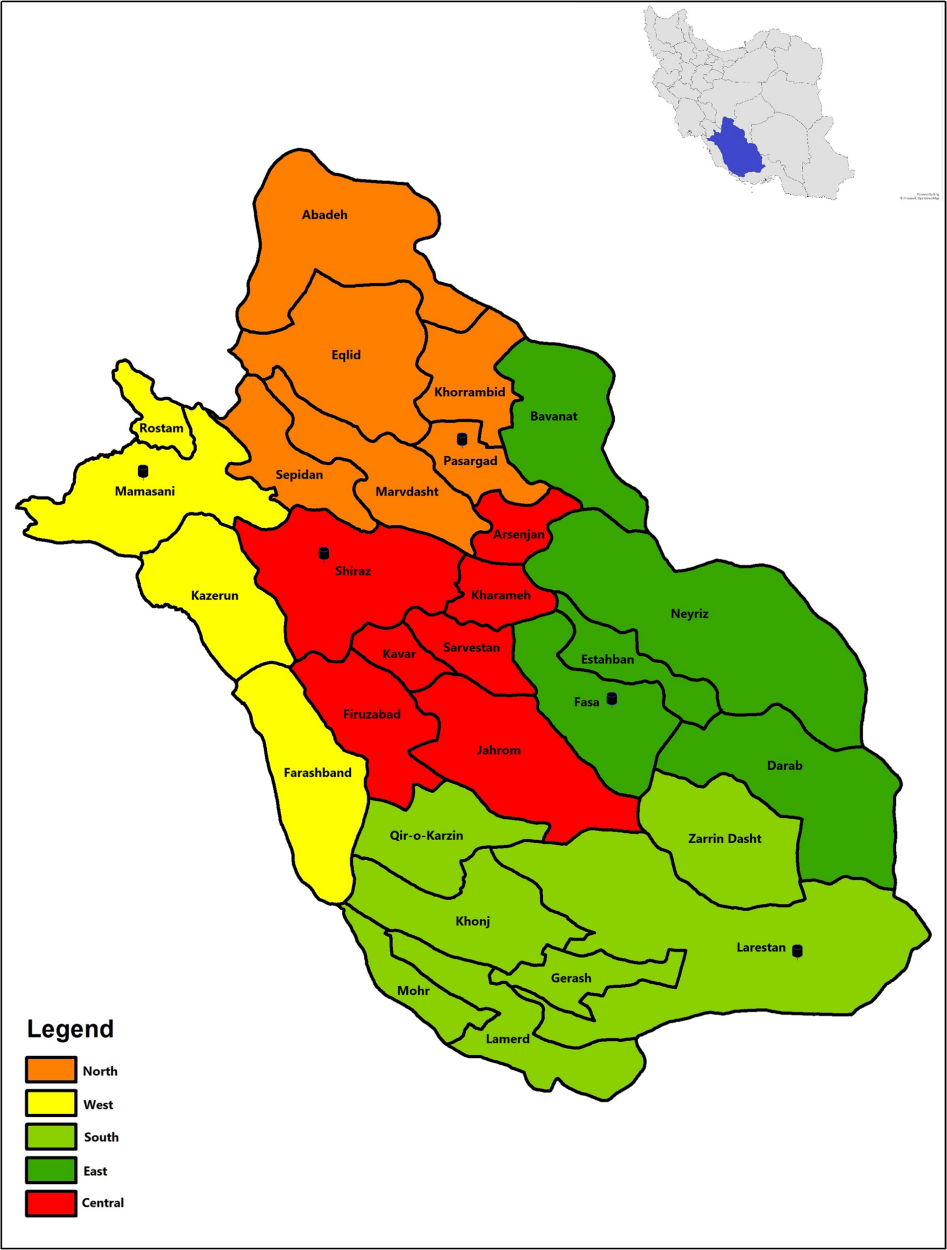
The map highlights the five districts of Fars province, from which the counties were selected

Cluster random sampling was employed to ensure a representative and diverse sample. Fars province, which is the 4th most populous province in Iran, has nearly 5 million inhabitants [22]. The province was stratified into five distinct districts: Central, West, East, North, and South. One county was randomly selected from each district: Shiraz (Central), Mamasani (West), Fasa (East), Pasargad (North), and Larestan (South) [Fig. 1]. Sample sizes were proportionally allocated based on the relative population of these counties. Participants for both case and control groups were drawn from the Health Information System (HIS) registry of each county, ensuring inclusion of both urban and rural populations. These registries are comprehensive databases maintained by the health network executive units including health houses and rural health centers, and contains detailed demographic and medical record information of the residents [23]. Within each county, participants were randomly selected. Controls were chosen from the same population as the cases to account for regional differences and for geographical matching, following a similar cluster random sampling approach. Supplementary Table 1 shows the distribution of participants across the selected counties.

Once potential participants were identified, they were contacted and screened for eligibility criteria, and those meeting the criteria were invited for face-to-face interview with our investigators present in the region. During these interviews, our researcher-developed data collection forms were utilized and filled out by the interviewers. We checked for significant differences in the age distributions between the two groups before finalizing the sampling. The age distribution of the case and control groups was compared across 10-year age intervals, and no statistically significant differences were found, as shown in Supplementary Table 2. This analysis ensured that the control group was appropriately age-matched to the case group. Standardized protocols were followed for participant interviews, prioritizing the most recent episode of SARS-CoV-2 infection in cases of multiple episodes. Interviewers were trained extensively to maintain consistency and minimize biases in participant responses, and participants were provided with detailed information about the study objectives before obtaining informed consent.

### Data collection

The current study utilized a researcher-designed data collection form to address demographic information, past health conditions, disease characteristics, and the impact of SARS‑CoV‑2 infection on the quality of life among adult patients. The primary conceptual framework of our data collection form was developed by extensive review of relevant literature [16, 17, 19, 24–28] and consultation with four public health experts to ensure comprehensive coverage of all necessary aspects of participants' medical history and pandemic-related symptoms. In addition, Longitudinal Population Studies SARS‑COV‑2 Questionnaire [24] and Symptom Burden Questionnaire for Long COVID [25] were utilized for designing items about disease symptoms in two prespecified phases of disease course and then underwent meticulous process of refinement and localization tailored to the specific characteristics of our targeted population. Later, Supplementary Online Patient Questionnaire [29] helped in designing items about demographics, social history and background health condition. The final adjustment of the data collection form was guided by incorporating cultural and contextual relevance, and local physicians’ insights to enhance its precision and applicability. Additionally, the face validity of the data collection form was reviewed and revised by two academic colleagues in the field of public health and epidemiology.

The data collection form designed for the SARS‑COV‑2 cases group encompassed 90 items covering sociodemographic information, past medical health conditions, smoking status, their course of SARS-CoV-2 infection, disease symptoms during the first 4 weeks of infection, medical care and supports received during and after the initial infection phase, duration of symptoms, and symptoms experienced more than one month post-infection. It also asked about the participants' perceived overall health status before the start of the pandemic and at the time of the interview. The data collection form developed for the control group comprised 49 items, mirroring the structural format of the data collection form for the case group. The control group data collection form omitted items specific to our case group and their infection course. Unlike the case group, which were queried about symptoms during both the initial infection phase and after 4 weeks, the control group were asked about symptoms already present in the SARS‑COV‑2 pandemic. The detailed data collection forms for both case and control groups, in both English and Persian, are provided as supplementary material for reference.

### Definition of variables

All definitions were adopted from standard Centers for Disease Control and Prevention (CDC) definitions, as outlined below:

1. Acute COVID-19 symptoms: symptoms experienced during the first four weeks of SARS-CoV-2 infection, which include, but are not limited to, fever, cough, shortness of breath, fatigue, muscle or body aches, loss of taste or smell, sore throat, congestion or runny nose, nausea or vomiting, and diarrhea [30]. These symptoms were self-reported by participants during interviews and recorded in the data collection form.
2. Body Mass Index (BMI): calculated by dividing a person's weight in kilograms by the square of their height in meters. BMI value is categorized into different groups according to standard ranges: Underweight (BMI less than 18.5), Normal weight (BMI between 18.5 and 24.9), Overweight (BMI between 25 and 29.9), and Obesity (BMI more than 30) [31].
3. Smoking status: A current smoker is defined as an adult who has smoked at least 100 cigarettes in their lifetime and is presently smoking. An ex-smoker refers to an adult who has smoked at least 100 cigarettes in their lifetime but has ceased smoking at the time of the interview. A never smoker is described as an adult who has either never smoked or has smoked fewer than 100 cigarettes in their lifetime [32].
4. LC: a broad spectrum of physical and mental health consequences persisting four or more weeks after initial infection with SARS‑COV‑2 [9]. The presence of LC was determined based on participants' self-reported symptoms that persisted or appeared after four weeks of COVID-19 contraction. These symptoms were categorized and recorded during the face-to-face interviews using our data collection form.

### Statistical analyses

Statistical analyses of collected data were performed using IBM SPSS statistical software version 23.0. Continuous variables were described by mean ± standard deviation (SD) or median, and categorical variables were expressed as the frequencies and relative frequencies along with their 95% confidence intervals (CIs). The statistical analyses were conducted using chi-square test. We calculated the age- and sex-standardized rate of having any symptoms for those with or without history of SARS-COV-2 using the World Health Organization standard population distribution 2000–2025 [33]. We conducted a binary logistic regression model to identify factors associated with LC in patients with a history of SARS‑COV‑2. Variables with a *p*-value of less than 0.3 in the univariate analyses were recruited for the analysis model. The crude and adjusted odds ratio (OR) were then calculated and reported. *P*-values less than 0.05 were considered of statistical significance.

## Results

A total of 2,010 participants were enrolled in this study, comprising 790 (39.3%) men and 1,220 (60.7%) women. Among them, 1,561 individuals (77.7%) had a previous history of SARS‑COV‑2 while 449 individuals did not recall being infected with SARS‑COV‑2.

Among the participants with a history of SARS-CoV-2 infection, 86.2% experienced only one episode of the infection. Among these cases, 915 patients (58.6%) contracted the infection before receiving any dose of vaccination. Furthermore, 1120 patients (71.8%) did not require hospitalization due to SARS-CoV-2. Among those hospitalized, 290 (18.6%) were admitted for less than 2 weeks, 151 (9.7%) for more than 2 weeks, and only 5 patients were hospitalized for over one month. Following the onset of the initial symptoms of SARS-CoV-2, a majority of individuals (59.4%) reported a return to normal functioning within the first one to two weeks. A smaller proportion (15.3%) reported a recovery period extending into the third or fourth week, while 4.5% indicated a recovery period exceeding one month. Notably, 20.8% of participants (325 individuals) reported no impact on their ability to work normally due to the infection.

The age of participants ranged from 18 to 89 years, with a mean age of 41.5 (± 11.1) years and a median of 41 years. The age distribution demonstrated that 53.4% of the total population falls within the 35–54 years age group. Furthermore, differences were noted in other demographic and clinical characteristics between these two patient groups, indicating significant associations between a history of SARS-CoV-2 infection and factors such as female gender, marital status, lower educational attainment, receiving fewer vaccine doses, or current smoking status. Further details regarding the demographic and clinical characteristics of the study participants are provided in Table 1.

**Table 1:** Demographic and clinical characteristics of study participants stratified by SARS‑COV‑2 history

Variable	Total population (n (%) or mean (± SD))	Patients without history of COVID-19 (n (%) or mean (± SD))	Patients with history of COVID-19 (n (%) or mean (± SD))	*P*-value
	2010 (100)	449 (100)	1561 (100)	
Age in years	41.54 ± 11.06	42.13 ± 11.92	41.37 ± 10.81	0.256
Gender				
Male	790 (39.3)	209 (46.5)	581 (37.2)	< 0.001
Female	1220 (60.7)	240 (53.5)	980 (62.8)	
Marital status				
Single	444 (22.1)	136 (30.3)	308 (19.7)	< 0.001
Married	1451 (72.2)	274 (61.0)	1177 (75.4)	
Divorced or widowed	115 (5.7)	39 (8.7)	76 (4.9)	
Educational level				
Illiterate	73 (3.6)	13 (2.9)	60 (3.8)	< 0.001
Primary school education	209 (10.4)	33 (7.3)	176 (11.3)	
Middle school or high school education	106 (5.3)	25 (5.6)	81 (5.2)	
High school diploma	1234 (61.4)	239 (53.2)	995 (63.7)	
Academic education	388 (19.3)	139 (31.0)	249 (16.0)	
Occupation				
Unemployed	223 (11.1)	58 (12.9)	165 (10.6)	< 0.001
Housewife	787 (39.2)	136 (30.3)	651 (41.7)	
Employed	896 (44.6)	227 (50.6)	669 (42.9)	
Retired	104 (5.2)	28 (6.2)	76 (4.9)	
Body mass index				
Underweight	27 (1.3)	4 (0.9)	23 (1.5)	0.007
Normal weight	527 (26.2)	91 (20.3)	436 (28.0)	
Overweight	1088 (54.2)	264 (58.8)	824 (52.9)	
Obese	366 (18.2)	90 (20.0)	276 (17.7)	
History of COVID-19 vaccination				
None	132 (6.6)	59 (13.1)	73 (4.7)	< 0.001
One dose of vaccination	252 (12.5)	19 (4.2)	233 (14.9)	
Two doses of vaccination	1084 (53.9)	167 (37.2)	917 (58.7)	
At least three doses of vaccination	542 (27.0)	204 (45.4)	338 (21.7)	
Smoking status				
Nonsmokers	1624 (80.8)	378 (84.2)	1246 (79.8)	0.001
Past smoker (stopped before COVID-19 outbreak)	52 (2.6)	17 (3.8)	35 (2.2)	
Past smoker (stopped after COVID-19 outbreak)	36 (1.8)	11 (2.4)	25 (1.6)	
Current smokers	298 (14.8)	43 (9.6)	255 (16.3)	
Having chronic lung disease	81 (4.0)	23 (5.1)	58 (3.7)	0.182
Having diabetes mellitus	127 (6.3)	38 (8.5)	89 (5.7)	0.034
Having cardiovascular disease	219 (10.9)	72 (16.0)	147 (9.4)	< 0.001
Having malignancy	12 (0.6)	6 (1.3)	6 (0.4)	0.021
Steroids/chemotherapy use	76 (3.8)	8 (1.8)	68 (4.4)	0.012

Table 2 presents a detailed overview of potential SARS‑COV‑2-related symptoms of various domains among the study participants, with frequencies being reported for individuals with history of SARS‑COV‑2 during and after the infection. For individuals without a history of SARS‑COV‑2, the prevalence of having any symptom was 23.4% (95% CI of 19.7%-27.5%). Those with a history of SARS‑COV‑2 had higher rates of having any symptoms during both the SARS‑COV‑2 and in the aftermath of SARS‑COV‑2 at 93.7% (95% CI of 92.3%-94.8%) and 36.4% (95% CI of 34.0%-38.8%), respectively. The age- and sex-standardized rates were 16.7% (95% CI of 14.6%-18.7%), 68.8% (95% CI of 68.0%-69.7%), and 29.0% (95% CI of 25.0%-32.9%), respectively. The probability of having any symptom was significantly higher among individuals with history of SARS‑COV‑2 after four weeks of the initial infection than those without history of SARS‑COV‑2 (*P*-value < 0.001). Subtracting the frequency of having symptoms in the participants without history of SARS‑COV‑2 from the frequency for those with history of SARS‑COV‑2 after the disease yielded crude and standardized rates of the presence of any symptoms attributable to SARS‑COV‑2 at 13% and 12.3%, respectively. The three predominant symptoms in the LC constellation, potentially attributed to SARS‑COV‑2, were challenges with sexual issues, fatigue, and digestive problems. Among participants with a history of SARS‑COV‑2 reporting symptoms during the infection, 59.5% (*n* = 870 individuals) noted a severe impact on their daily life due to the symptoms. Among those with any symptoms after four weeks of the initial infection, however, the majority (78.0%) reported a mild to moderate impact, with only 12.7% experiencing a severe impact on their daily life.

**Table 2:** Comprehensive overview of potential SARS‑COV‑2-related symptoms among study participants with and without history of SARS‑COV‑2

Category of symptoms	Symptoms	Individuals without history of COVID-19 n (%: 95% CI)	Individuals with history of COVID-19	
			During COVID-19 n (%: 95% CI)	After COVID-19 n (%: 95% CI)
		449 (100)	1561 (100)	
Respiratory	Shortness of breath	3 (0.7: 0.2–2.1)	323 (20.7: 18.8–22.8)	68 (4.4: 3.4–5.5)
	Experiencing accelerated breathing or waking up at night due to a sensation of breathlessness	9 (2.0: 1.0–3.8)	382 (24.5: 22.4–26.7)	105 (6.7: 5.6–8.1)
Circulatory	Palpitations, lightheadedness or dizziness when standing, swelling in extremities, as well as in the face, lips, tongue, and throat, or cold extremities lasting longer than usual or with increased coldness	15 (3.3: 2.0–5.5)	385 (24.7: 22.6–26.9)	126 (8.1: 6.8–9.5)
Fatigue	Tiredness and fatigue (characterized by a feeling of physical or mental exhaustion that does not alleviate with rest, and a worsening of symptoms after engaging in simple physical or mental activities)	16 (3.6: 2.2–5.7)	886 (56.8: 54.3–59.2)	186 (11.9: 10.4–13.6)
Cognitive	Brain fog (feeling sluggish, jet-lagged, or blanking out), confusion, memory loss, difficulty concentrating or planning, or challenges in finding the right words	2 (0.4: 0.1–1.8)	156 (10.0: 8.6–11.6)	34 (2.2: 1.6–3.0)
	Difficulty in comprehending others' speech, slurred speech, or reading challenges unrelated to dyslexia	0 (0.0)	80 (5.1: 4.1–6.3)	16 (1.0: 0.6–1.7)
Mobility	Tremors, difficulty maintaining balance, and challenges with movement and coordination, including uncontrollable shaking or trembling in a specific part of the body	1 (0.2: 0.03–1.6)	459 (29.4: 27.2–31.7)	53 (3.4: 2.6–4.4)
Sleep	Difficulties falling asleep, experiencing changes in sleep duration-either sleeping shorter or longer than usual-and interruptions during sleep	0 (0.0)	660 (42.3: 39.8–44.8)	93 (6.0: 4.9–7.2)
Ear, nose, and throat	Earache, tinnitus, heightened sensitivity to sounds, or new-onset hearing loss	4 (0.9: 0.3–2.4)	494 (31.6: 29.4–34.0)	100 (6.4: 5.3–7.7)
	Altered sense of smell or taste, sneezing, runny nose, or sinus congestion, characterized by discomfort or a feeling of 'fullness' around the nose, cheeks, forehead, or eyes	7 (1.6: 0.7–3.2)	853 (54.6: 52.2–57.1)	106 (6.8: 5.6–8.2)
	Production of mucus, cough, sore throat, hoarse voice, difficulty swallowing, mouth ulcers, dry mouth, or a worsening of known dental problems	17 (3.8: 2.4–6.0)	1002 (64.2: 61.8–66.5)	159 (10.2: 8.8–11.8)
Digestive	Abdominal pain, bloating, nausea, indigestion, heartburn, diarrhea, or constipation	11 (2.4: 1.3–4.4)	906 (58.0: 55.6–60.5)	158 (10.1: 8.7–11.7)
	Unintended weight loss or weight gain	15 (3.3: 2.0–5.5)	335 (21.5: 19.5–23.6)	37 (2.4: 1.7–3.3)
Musculoskeletal	Muscle pain, weakness, or stiffness, muscle twitching or cramping, tingling, or numbness	16 (3.6: 2.2–5.7)	890 (57.0: 54.5–59.5)	155 (9.9: 8.5–11.5)
	Joint pain, swelling, or stiffness	9 (2.0: 1.0–3.8)	802 (51.4: 48.9–53.9)	140 (9.0: 7.6–10.5)
	Chest pain, pain during breathing, stabbing or burning sensations in various parts of the body, overall body aches, or headaches	7 (1.6: 0.7–3.2)	412 (26.4: 24.3–28.6)	114 (7.3: 6.1–8.7)
Psychological	Lack of interest, sadness, mood swings, loneliness, changes in appetite, a sense of hopelessness about the future, and anxiety	2 (0.4: 0.1–1.8)	787 (50.4: 47.9–52.9)	109 (7.0: 5.8–8.4)
	Experiencing thoughts about self-harm and a sense of not being the person one was before the disease	5 (1.1: 0.4–2.7)	188 (12.0: 10.5–13.8)	13 (0.8: 0.5–1.4)
Dermatological	Dry or itchy skin	1 (0.2: 0.03–1.6)	387 (24.8: 22.7–27.0)	77 (4.9: 4.0–6.1)
	Purple-red spots on the feet, rashes, or hives	4 (0.8: 0.3–2.4)	186 (11.9: 10.4–13.6)	27 (1.7: 1.2–2.5)
	Hair loss, or changes to the nails (ridging, pitting, discoloration, or brittle nails)	12 (2.7: 1.5–4.7)	447 (28.6: 26.4–30.9)	93 (6.0: 4.9–7.2)
Ocular	Red, dry, itchy, or watery eyes, pressure behind the eyes, flashing lights, or a sensation of a foreign body	3 (0.6: 0.2–2.1)	482 (30.9: 28.6–33.2)	46 (2.9: 2.2–3.9)
	Pain behind the eyes, blurred vision, double vision (not related to wearing glasses), or sensitivity to light	3 (0.6: 0.2–2.1)	712 (45.6: 43.2–48.1)	19 (1.2: 0.8–1.9)
Reproductive and sexual	In females: Changes in menstrual periods, exacerbation of premenstrual symptoms, increased occurrence of blood clots, or concerns about the ability to have an orgasm In males: Decreased interest in sexual contact or difficulties with ejaculation	5 (1.1: 0.4–2.7)	355 (22.7: 20.7–24.9)	95 (6.1: 5.0–7.4)
	In females: Vaginal dryness, or discharge, or decreased interest in sex In males: Challenges related to maintaining an erection	4 (0.8: 0.3–2.3)	753 (48.2: 45.8–50.7)	159 (10.2: 8.8–11.8)
Urological	Loss of control of urine (leakage), difficulty passing urine, increased thirst, or frequency of urination	7 (1.6: 0.7–3.2)	91 (5.8: 4.8–7.1)	17 (1.2: 0.7–1.7)
Immune	Heightened reaction to known or new allergies	18 (4.0: 2.5–6.3)	286 (18.3: 16.5–20.3)	94 (6.0: 4.9–7.3)
Other symptoms	Fever or chills	2 (0.4: 0.1–1.8)	885 (56.7: 54.2–59.1)	52 (3.3: 2.5–4.3)
	Sweating problems, hot flushes, swelling of lymph nodes, or vertigo, characterized by a sensation of spinning that affects the balance	26 (5.8: 4.0–8.4)	925 (59.3: 56.8–61.7)	109 (7.0: 5.8–8.4)
Composite outcome of the presence of any symptom		105 (23.4: 19.7–27.5)	1462 (93.7: 92.3–94.8)	568 (36.4: 34.0–38.8)

The results presented in Table 3 elucidate the variables correlated with the occurrence of LC symptoms among individuals previously infected with SARS-COV-2. Notably, age exhibited a substantial correlation, with individuals aged 55 years and older displaying increased likelihood of experiencing LC symptoms (OR of 2.10, 95% CI of 1.29–3.40, *P*-value = 0.003) compared to those aged 18–34 years. Additionally, several other factors demonstrated significant associations with the manifestation of LC symptoms, including prior hospitalization due to SARS-COV-2, both for durations of less than and more than 2 weeks, the presence of cardiovascular disease, and the utilization of steroid or chemotherapy regimens. Furthermore, our findings indicate that vaccination against COVID-19, whether partial or complete, does not significantly associate with development of Long COVID (LC) symptoms among those with a history of COVID-19.

**Table 3:** Factors associated with the presence of any LC symptoms among the participants with COVID-19 history

Variable	Crude OR (95% CI)	*P* value	Adjusted OR (95% CI)	*P* value
Age groups (Ref: 18–34 years)				
35–54 years	1.21 (0.95–1.54)	0.130	1.08 (0.78–1.49)	0.652
55 years and above	3.09 (2.23–4.29)	< 0.001	2.10 (1.29–3.40)	0.003
Marital status (Ref: Single)				
Married	1.31 (1.00–1.71)	0.049	1.03 (0.67–1.58)	0.908
Divorced or widowed	1.34 (0.80–2.26)	1.11	0.58 (0.29–1.16)	0.127
Educational level (Ref: Illiterate)				
Primary school education	0.54 (0.30–0.99)	0.045	0.67 (0.34–1.34)	0.258
Middle school or high school education	0.61 (0.31–1.19)	0.149	1.30 (0.58–2.92)	0.517
High school diploma	0.27 (0.16–0.47)	< 0.001	0.60 (0.31–1.16)	0.127
Academic education	0.46 (0.26–0.82)	0.009	0.92 (0.46–1.86)	0.820
Occupation (Ref: Unemployed)				
Housewife	1.25 (0.87–1.79)	0.229	1.11 (0.64–1.90)	0.714
Employed	1.07 (0.74–1.54)	0.715	0.81 (0.50–1.33)	0.415
Retired	2.17 (1.24–3.77)	0.006	0.54 (0.25–1.15)	0.109
Body mass index (Ref: Normal weight)				
Overweight	1.38 (1.07–1.77)	0.012	1.24 (0.94–1.64)	0.136
Obese	1.81 (1.32–2.48)	< 0.001	1.28 (0.89–1.85)	0.179
Smoking status (Ref: Nonsmoker)				
Past smoker	2.11 (1.25–3.55)	0.005	1.54 (0.83–2.83)	0.168
Current smoker	0.76 (0.57–1.02)	0.065	0.96 (0.66–1.39)	0.823
History of hospitalization for SARS‑CoV‑2 (Ref: None)				
Less than 2 weeks of hospitalization	2.95 (2.26–3.84)	< 0.001	2.75 (2.07–3.66)	< 0.001
More than 2 weeks of hospitalization	4.30 (3.01–6.13)	< 0.001	3.09 (2.06–4.64)	< 0.001
History of SARS‑CoV‑2 vaccination (Ref: None)				
One dose of vaccination	1.68 (0.96–2.96)	0.070	1.68 (0.90–3.15)	0.104
Two doses of vaccination	1.03 (0.61–1.73)	0.913	1.01 (0.56–1.81)	0.975
At least three doses of vaccination	2.24 (1.30–3.85)	0.004	1.78 (0.97–3.24)	0.061
Chronic lung disease (Ref: None)	2.22 (1.31–3.77)	0.003	1.01 (0.55–1.85)	0.980
Diabetes mellitus (Ref: None)	2.25 (1.46–3.46)	< 0.001	0.87 (0.51–1.48)	0.608
Cardiovascular disease (Ref: None)	3.30 (2.32–4.69)	< 0.001	1.54 (1.00–2.36)	0.048
Malignancy (Ref: None)	3.51 (0.64–19.25)	0.147	1.26 (0.21–7.53)	0.798
Steroids/chemotherapy use (Ref: No use)	6.15 (3.48–10.89)	< 0.001	2.23 (1.12–4.43)	0.023

## Discussion

The findings of our investigation yield insights into the enduring effects of SARS-COV-2, offering novel perspectives and avenues for future research in this domain. Our study uncovers a significant prevalence of LC, affecting 36.4% of our patient cohort. Among these cases, prevalent manifestations include fatigue, upper respiratory tract symptoms such as sino-nasal affections and altered sense of smell or taste, sexual well-being concerns, gastrointestinal disturbances, and musculoskeletal symptoms. Our results underscore the multifaceted nature of LC and unveil a complex array of enduring manifestations stemming from SARS-COV-2 infection. Sexual health symptoms including decreased libido, erectile dysfunction, and vaginal dryness, often underestimated in many surveys assessing LC symptomatology, emerged as a prominent aspect in our study. Research has demonstrated that factors such as hypothalamic-pituitary–gonadal axis dysfunction, endothelial dysfunction, and psychological distress play significant roles in contributing to these sexual health problems [34–36]. These symptoms not only ranked among the top three most frequently observed manifestations during the LC course in our patients but also demonstrated a substantial increase from 0.8% in non-COVID participants to 10.2% in LC patients. Given the significant prevalence of sexual functioning problems in our cases, we encourage researchers to acknowledge their importance and explore this domain more comprehensively.

Moreover, the findings elucidate predisposing factors that increase the likelihood of our patients developing LC following the acute phase of infection. Notably, age emerges as a significant determinant, with individuals aged 55 and older exhibiting elevated odds of enduring symptoms compared to their counterparts in the 18–34 age bracket. This observation may be attributed to immune-senescence among individuals aged 55 and above, compounded by a higher prevalence of chronic ailments, potentially exacerbated by the repercussions of pandemics and factors such as social isolation and inadequate support [26, 37]. In addition, we found individuals with a history of SARS‑COV‑2-related hospitalization, those with pre-existing cardiovascular comorbidities, and individuals receiving steroids or chemotherapy regimens are more likely to experience LC symptoms. Importantly, this increased likelihood was more pronounced in individuals with a hospital stay lasting more than two weeks. Previous studies have also showed that patients with a history of hospital admission tend to undergo a prolonged course of LC, with dyspnea, fatigue, and sleep disturbances being more prevalent in this group compared to non-hospitalized patients. [27, 28]. In addition, studies have revealed that within the range of symptoms associated with LC, certain specific symptoms can significantly indicate the likelihood of persistent symptoms. For instance, in a prospective cohort study of confirmed COVID-19 cases, the presence of dyspnea by day 10 and fatigue by day 60 after contracting COVID-19 was found to increase the risk of developing LC by almost double and triple, respectively [38].

Despite a substantial proportion of our participants having received COVID-19 vaccines, our data did not support a statistically significant difference in the prevalence of LC symptoms between vaccinated and unvaccinated individuals. Previous literature suggests that vaccination either reduces the risk or has a neutral effect on the development of LC [39]. However, inconsistent findings and a high degree of inter-study heterogeneity preclude a robust meta-analysis [39–41]. The protection observed in some studies may be attributed to the vaccine's effectiveness in preventing severe forms of acute infection and subsequent hospitalization, which are significant risk factors for LC [41]. Studies have shown that breakthrough SARS-CoV-2 infections in vaccinated individuals are associated with lesser degrees of uncoordinated responses from the adaptive immune system [42]. COVID-19 vaccines accelerate viral clearance, counter virus-induced humoral memory immunity, and reduce the formation of autoantibodies [43, 44]- processes that may contribute to the pathogenesis of LC. Additionally, there are some evidence that COVID-19 vaccine uptake may lead to potential symptom improvement and increased rates of remission in unvaccinated patients with LC [45, 46]. Further research is needed to clarify the role of vaccination in preventing LC and potentially improving outcomes in affected individuals.

In view of the existing literature on long-term SARS‑COV‑2 effects, our findings align with prior studies emphasizing a diverse array of symptoms persisting beyond the acute phase. Based on a meta-analysis including 31 studies on LC, its pooled prevalence has been estimated 43% (95% CI: 39, 46). As implied by the authors, there is a vast heterogeneity across included studies, including variations in study methodologies, different included symptoms and patient population [18]. Discrepancies in LC definition emerge as a significant determinant contributing to the observed heterogeneity. CDC characterizes it as a broad spectrum of physical and mental health consequences persisting four or more weeks after initial infection, while, the World Health Organization defines it as the continuation or emergence of new symptoms twelve weeks post-initial infection, lasting at least two months without alternative explanations [9, 47]. In addition, as illustrated in the aforementioned study, other than these two definitions of LC based on the interval to the index date, 23 out of the total 31 included studies, have assessed lingering symptoms of SARS‑COV‑2 at 60 or 120 days after index date [18]. This temporal variability in symptoms evaluation poses a challenge in understanding the trajectory and persistence of symptoms, highlighting the need for standardized criteria to enhance comparability between studies.

Furthermore, a notable limitation observed across a majority of studies pertains to the absence of control groups in the evaluation of persistent symptoms. It appears that the assessment of psychomotor symptoms and health status may be prone to overestimation, as many questionnaires commonly include numerous symptoms prevalent in the general population, rendering them nonspecific to SARS-CoV-2 infection. To mitigate this limitation, comparing our cases with individuals who have not been infected with SARS-COV-2 could offer a clearer understanding of the true impact of the virus on psychomotor symptoms and overall health. In this context, a prospective questionnaire-based study involving 76,422 participants matched with their respective controls revealed that only 12% of patients exhibited new or persistent symptoms at 12 weeks following the index date, which could be attributed to their initial infection [19]. This significant contrast with previously estimated percentages of LC in existing literature highlights the importance of correction for pre-existing symptoms in case group as well as symptomatic conditions in control group.

In our studies, as well, 23.4% of our control group had at least one of core symptoms designed in the data collection form. Taking the pre-COVID health status of our cases into account would provide a valuable benchmark in interpreting LC symptoms and prevalence. Regarding this, if we consider our control group as an indicator of health status before SARS‑CoV‑2 infection, given the LC prevalence of 36.4%, as a result, only 13% would be specifically attributed to SARS‑COV‑2's long-term impacts. A recent systematic review and meta-analysis on the long-term health effects of SARS‑COV‑2 revealed that only 11.3% of included studies (22 out of 194), even without excluding those with inappropriately matched controls, recruited comparator groups [26]. The absence of a control group complicates comparisons regarding the burden of LC and symptom profiles between individuals with and without history of SARS‑CoV‑2 infection, as some individuals may develop these non-specific manifestations due to background health conditions or due to broader impacts of the pandemic [48]. In this regard, investigations revealed increased rates of somatic symptoms across the general population during the pandemic [49].

While our study offers valuable insights into the long-term repercussions of SARS-COV-2, several limitations need to be acknowledged. Our investigation relied on self-reported data collection. Despite endeavors to mitigate bias through clear instructions and structured protocols, it is imperative to recognize this limitation and interpret the findings with consideration of the potential influence of recall bias on the study outcomes. Additionally, a substantial proportion of missing data regarding the approximate date of SARS-COV-2 contraction was noted during interviews, which impeded our ability to explore temporal patterns of LC and to investigate the progression of symptoms over time. Furthermore, the cross-sectional nature of the study design precludes the establishment of causation. Future research endeavors employing larger and more diverse cohorts, alongside longitudinal study designs, would augment the robustness of our findings.

## Conclusions

In conclusion, investigation illuminates the intricate landscape of LC and its symptom profiles. The results underscore a high prevalence of LC among individuals with a prior history of SARS-CoV-2 infection. The diverse array of symptoms observed in our study aligns with the expanding body of evidence highlighting the heterogeneous nature of LC. We strongly advocate for the inclusion of well-matched control groups and standardized diagnostic criteria to enhance our understanding of LC and improve patient care. This approach is crucial for reducing the risk of misdiagnosing other conditions as LC. This study serves as a foundational step, identifying avenues for further exploration and intervention aimed at alleviating the enduring impact of SARS-CoV-2 on individuals' health and well-being.

### Supplementary Information

Additional file 1:

## Data Availability

The datasets used during this study are available on reasonable request to corresponding author.

## Abbreviations

BMI: Body Mass Index
CDC: Centers for Disease Control and Prevention
COVID-19: Coronavirus disease 2019
LC: Long COVID-19
SARS-CoV-2: Severe acute respiratory syndrome coronavirus 2

## References

[1] CDC. *COVID-19 Timeline*. Last Reviewed: March 15, 2023 [Cited March 6, 2024]; Available from: https://www.cdc.gov/museum/timeline/covid19.html.

[2] Cascella M, R.M., Aleem A, et al. *Features, Evaluation, and Treatment of Coronavirus (COVID-19)* [Updated 2023 Aug 18]; Available from: https://www.ncbi.nlm.nih.gov/books/NBK554776/. 32150360

[3] WHO. *Number of COVID-19 cases reported to WHO (cumulative total)*. [Cited December 25, 2022]; Available from: https://data.who.int/dashboards/covid19/cases.

[4] IHME. *COVID-19 Results Briefing*. January 21, 2022 [Cited December 21, 2022]; Available from: https://www.healthdata.org/sites/default/files/files/1_briefing_Global_5.pdf.

[5] Marco Cascella, M.R., Abdul Aleem, Scott C. Dulebohn, Raffaela Di Napoli, *Features, Evaluation**, **and Treatment of Coronavirus (COVID-19)*. Last Update: August 18, 2023, StatPearls Publishing: StatPearls [Internet]. Treasure Island (FL). 32150360

[6] Heidari M, Sayfouri N, Jafari H. Consecutive Waves of COVID-19 in Iran: Various Dimensions and Probable Causes. Disaster Med Public Health Prep. 2022;17:e136. 35152937 10.1017/dmp.2022.45 PMC9509792

[7] Shirafkan H, et al. Demographics, clinical characteristics, and outcomes in hospitalized patients during six waves of COVID-19 in Northern Iran: a large cohort study. Sci Rep. 2023;13(1):22527. 38110656 10.1038/s41598-023-50139-8 PMC10728067

[8] Worldometer. *Total Coronavirus Cases in Iran*. April 13, 2024; Available from: https://www.worldometers.info/coronavirus/country/iran/#google_vignette.

[9] CDC. *Post-COVID Conditions: Overview for Healthcare Providers*. Updated Sept. 11, 2023 [Cited December 21, 2022]; Available from: https://www.cdc.gov/coronavirus/2019-ncov/hcp/clinical-care/post-covid-conditions.html.

[10] Yin, K., et al., *Long COVID manifests with T cell dysregulation, inflammation and an uncoordinated adaptive immune response to SARS-CoV-2.* Nature Immunology, 2024. 10.1038/s41590-023-01724-6 PMC10834368 38212464

[11] Yang C, et al. Association of SARS-CoV-2 infection and persistence with long COVID. Lancet Respir Med. 2023;11(6):504–6. 37178694 10.1016/S2213-2600(23)00142-X PMC10171832

[12] Rojas M, et al. Autoimmunity is a hallmark of post-COVID syndrome. J Transl Med. 2022;20(1):129. 35296346 10.1186/s12967-022-03328-4 PMC8924736

[13] Castanares-Zapatero D, et al. Pathophysiology and mechanism of long COVID: a comprehensive review. Ann Med. 2022;54(1):1473–87. 35594336 10.1080/07853890.2022.2076901 PMC9132392

[14] Sabioni LR, et al. Systemic microvascular dysfunction in COVID-19. Am J Cardiovasc Dis. 2020;10(4):386–91. 33224588 PMC7675177

[15] Tsampasian V, et al. Risk Factors Associated With Post−COVID-19 Condition: A Systematic Review and Meta-analysis. JAMA Intern Med. 2023;183(6):566–80. 36951832 10.1001/jamainternmed.2023.0750 PMC10037203

[16] Sadat Larijani M, et al. Characterization of long COVID-19 manifestations and its associated factors: A prospective cohort study from Iran. Microb Pathog. 2022;169. 35690233 10.1016/j.micpath.2022.105618 PMC9176176

[17] Asadi-Pooya AA, et al. Long COVID in children and adolescents. World J Pediatr. 2021;17(5):495–9. 34478045 10.1007/s12519-021-00457-6 PMC8414448

[18] Chen C, et al. Global Prevalence of Post-Coronavirus Disease 2019 (COVID-19) Condition or Long COVID: A Meta-Analysis and Systematic Review. J Infect Dis. 2022;226(9):1593–607. 35429399 10.1093/infdis/jiac136 PMC9047189

[19] Ballering AV, et al. Persistence of somatic symptoms after COVID-19 in the Netherlands: an observational cohort study. The Lancet. 2022;400(10350):452–61. 10.1016/S0140-6736(22)01214-4 PMC9352274 35934007

[20] Charan J, Biswas T. How to calculate sample size for different study designs in medical research? Indian J Psychol Med. 2013;35(2):121–6. 24049221 10.4103/0253-7176.116232 PMC3775042

[21] Pourhoseingholi MA, Vahedi M, Rahimzadeh M. Sample size calculation in medical studies. Gastroenterol Hepatol Bed Bench. 2013;6(1):14–7. 24834239 PMC4017493

[22] *The population of Iran's provinces at a glance*. 29 May, 2022 [Cited February 16, 2024]; Available from: https://amar.org.ir/news/ID/17399/جمعیت-استان-های-ایران-در-یک-نگاه.

[23] Leila Doshmangir, M.B., Reza Majdzadeh, And Amirhossein Takian, *So Near, So Far: Four Decades of Health Policy Reforms in Iran, Achievements and Challenges.* Archives of Iranian Medicine, 22 October, 2019. 31679362

[24] breistol, U.o. *The Wellcome Covid-19 Questionnaire*. June 2021; Available from: https://www.bristol.ac.uk/alspac/covid-19/wellcome-covid-19/.

[25] Hughes SE, et al. Development and validation of the symptom burden questionnaire for long covid (SBQ-LC): Rasch analysis. BMJ. 2022;377. 35477524 10.1136/bmj-2022-070230 PMC9043395

[26] O’Mahoney LL, et al. The prevalence and long-term health effects of Long Covid among hospitalised and non-hospitalised populations: A systematic review and meta-analysis. EClinicalMedicine. 2023;55. 36474804 10.1016/j.eclinm.2022.101762 PMC9714474

[27] Pérez-González A, et al. Long COVID in hospitalized and non-hospitalized patients in a large cohort in Northwest Spain, a prospective cohort study. Sci Rep. 2022;12(1):3369. 35233035 10.1038/s41598-022-07414-x PMC8888560

[28] Yuan, N., et al., *Post-acute COVID-19 symptom risk in hospitalized and non-hospitalized COVID-19 survivors: A systematic review and meta-analysis.* 2023. **11**. 10.3389/fpubh.2023.1112383 PMC9978404 36875356

[29] *Online Supplementary materials, patient questionnaire*. [cited 2023 march 16]; Available from: https://www.dovepress.com/get_supplementary_file.php?f=316186.doC.

[30] CDC. *Symptoms of COVID-19*. Updated March 15, 2024 [cited July 5, 2024]; Available from: https://www.cdc.gov/coronavirus/2019-ncov/symptoms-testing/symptoms.html.

[31] CDC. *Defining Adult Overweight & Obesity*. Last Reviewed: June 3, 2022 [Cited December 25, 2022]; Available from: https://www.cdc.gov/obesity/basics/adult-defining.html.

[32] CDC. *National Center for Health Statistics*. Last reviewed: August 29, 2017 [Cited December 25, 2022]; Available from: https://www.cdc.gov/nchs/nhis/tobacco/tobacco_glossary.htm#:~:text=Former%20smoker%3A%20An%20adult%20who,at%20the%20time%20of%20interview.

[33] Ahmad, O.B., C. Boschi Pinto, and A.D. Lopez, *Age Standardization of Rates**: **A New WHO Standard.* GPE Discussion Paper Series: No 31, 2001: p. 10–12.

[34] Al-kuraishy HM, et al. Long COVID and risk of erectile dysfunction in recovered patients from mild to moderate COVID-19. Sci Rep. 2023;13(1):5977. 37045862 10.1038/s41598-023-32211-5 PMC10092929

[35] Bakr AM, El-Sakka AI. Erectile dysfunction among patients and health care providers during COVID-19 pandemic: A systematic review. Int J Impot Res. 2022;34(2):145–51. 34992226 10.1038/s41443-021-00504-w

[36] Zhang J, et al. Prevalence and risk factors of erectile dysfunction in COVID-19 patients: a systematic review and meta-analysis. J Endocrinol Invest. 2023;46(4):795–804. 36307637 10.1007/s40618-022-01945-w PMC9616422

[37] Mansell V, et al. Long COVID and older people. Lancet Healthy Longev. 2022;3(12):e849–54. 36480981 10.1016/S2666-7568(22)00245-8

[38] Abu Hamdh, B. and Z. Nazzal, *A prospective cohort study assessing the relationship between long-COVID symptom incidence in COVID-19 patients and COVID-19 vaccination.* Scientific Reports, 2023. **13**(1): p. 4896. 10.1038/s41598-023-30583-2 PMC10039348 36966161

[39] Byambasuren O, et al. Effect of covid-19 vaccination on long covid: systematic review. BMJ Med. 2023;2(1):e000385. 36936268 10.1136/bmjmed-2022-000385 PMC9978692

[40] Watanabe A, et al. Protective effect of COVID-19 vaccination against long COVID syndrome: A systematic review and meta-analysis. Vaccine. 2023;41(11):1783–90. 36774332 10.1016/j.vaccine.2023.02.008 PMC9905096

[41] Ceban F, et al. COVID-19 vaccination for the prevention and treatment of long COVID: A systematic review and meta-analysis. Brain Behav Immun. 2023;111:211–29. 36990297 10.1016/j.bbi.2023.03.022 PMC10067136

[42] Chia PY, et al. Virological and serological kinetics of SARS-CoV-2 Delta variant vaccine breakthrough infections: a multicentre cohort study. Clin Microbiol Infect. 2022;28(4):612.e1–612.e7. 34826623 10.1016/j.cmi.2021.11.010 PMC8608661

[43] Mishra, P.K., et al., *Vaccination boosts protective responses and counters SARS-CoV-2-induced pathogenic memory B cells.* medRxiv, 2021.

[44] Li H, et al. Vaccination reduces viral load and accelerates viral clearance in SARS-CoV-2 Delta variant-infected patients. Ann Med. 2023;55(1):419–27. 36862600 10.1080/07853890.2023.2166681 PMC9991402

[45] Grady, C.B., et al., *Impact of COVID-19 vaccination on symptoms and immune phenotypes in vaccine-naïve individuals with Long COVID.* medRxiv, 2024. 10.1038/s43856-025-00829-3 PMC12064684 40346201

[46] Edwards F, Hamilton FW. Impact of covid-19 vaccination on long covid. BMJ Medicine. 2023;2(1). 36936263 10.1136/bmjmed-2022-000470 PMC9978666

[47] WHO. *Post COVID-19 condition (Long COVID)*. 7 December 2022 [Cited December 25, 2022]; Available from: https://www.who.int/europe/news-room/fact-sheets/item/post-covid-19-condition#:~:text=It%20is%20defined%20as%20the,months%20with%20no%20other%20explanation.

[48] Tracy Beth H, Shamez L, Vinay P. How methodological pitfalls have created widespread misunderstanding about long COVID. BMJ Evid Based Med. 2024;29(3):142–6. 10.1136/bmjebm-2023-112338 PMC11137465 37748921

[49] Theocharis A, Antonopoulos V, Christodoulou NG. Somatic symptoms associated with mental distress during the COVID-19 pandemic: a systematic review. Australas Psychiatry. 2023;31(2):147–56. 36825513 10.1177/10398562231156380 PMC9969186

