# Supplementary material for: Epidemiological and clinical characteristics of long COVID-19 among Iranians: A community-based study in southern Iran
Source: BMC Public Health. 2024 Jul 26;24:2007. doi: 10.1186/s12889-024-19543-1 (PMC11282730; doi:10.1186/s12889-024-19543-1)
Supplement: Supplementary file 1 — Additional file 1: [file 12889_2024_19543_MOESM1_ESM.pdf]

# Supplementary Tables

Supplementary Table 1. The distribution of participants across the selected counties.

| Selected counties | Patients without history of COVID-19<br>n (%)<br>N = 449 | Patients with history of COVID-19<br>n (%)<br>N = 1561 | Total population of counties |
|---|---|---|---|
| Shiraz | 307 (68.3) | 1227 (78.6) | 1,869,001 |
| Mamasani | 39 (8.6) | 100 (6.4%) | 323,434 |
| Larestan | 50 (11.1) | 102 (6.5) | 213,920 |
| Fasa | 44 (9.7) | 110 (7.0) | 205,187 |
| Pasargad | 9 (2.0) | 22 (1.4) | 30,118 |

Supplementary Table 2. Age distribution of patients with and without a history of COVID-19 and comparison of proportions p-values.

| 10-year interval age categories | Patients without history of COVID-19<br>n (%)<br>N = 449 | Patients with history of COVID-19<br>n (%)<br>N = 1561 | Comparison of proportions p-value |
|---|---|---|---|
| 18-29 | 81 (18.0) | 223 (14.3) | 0.0539 |
| 30-39 | 124 (27.6) | 30.5 (476) | 0.2368 |
| 40-49 | 120 (26.7) | 492 (31.5) | 0.0515 |
| 50-59 | 90 (20.0) | 288 (18.4) | 0.4442 |
| 60-70 | 32 (7.1) | 77 (4.9) | 0.0690 |
| >70 | 2 (0.4) | 5 (0.3) | 0.7419 |

**English Version of Data Collection Form-For Cases**

**COVID-19 CASES (GROUP 1)**

**Best condition (No disease or condition in items 8 to 13, 19 to 48, 60 to 89) is 1.**

**Personal information:**

1) **Age**: ……. / 2) **Sex**: Male☐1 Female☐2 / 3) **Weight**: ……. / 4) **Height**: …….
5) **Level of Education**: Illiterate ☐1 Less than 6th grade ☐2 Less than high school diploma ☐3 high school diploma ☐4 Technician degree ☐5 Bachelor or master degree ☐6 Doctorate degree ☐7

6) **Employment status**: Employed☐1 Unemployed☐2 Housewife☐3 Post-High School student☐4 Retired☐5

7) **Marital status**: Single☐1 Married☐2 Divorced☐3 Widow☐4

**Did you already have any of the following health problems or conditions since before COVID-19 pandemic or before being infected with COVID-19?** ***(Tick all that apply)***

| | 1 | 2 |
|---|---|---|
| **8)** Lung diseases | No | Yes |
| **9)** Diabetes | No | Yes |
| **10)** Cardiovascular diseases (ex: heart failure, kidney diseases, hypertension) | No | Yes |
| **11)** Autoimmune diseases (ex: SLE, rheumatoid arthritis) | No | Yes |
| **12)** Malignancy (any type) | No | Yes |
| **13)** Medication (corticosteroids, chemotherapy) | No | Yes |

**14) What is your Smoking status?**
1)Non-Smoker
2) Ex-Smoker (stopped before COVID-19 outbreak)
3)Ex-Smoker (stopped after COVID-19 outbreak)
4) Current smoker

**15) Do you think that you currently have or have ever had COVID-19?**
1)No
2) Yes, confirmed by a positive test (PCR, rapid test)
3)Yes, based on medical advice
4) Yes, based on strong personal suspicion (close contact)

**16) Do you think you have caught COVID-19 more than once?**
1)No
2) Yes, confirmed by a positive test
3)Yes, based on medical advice
4) Yes, based on strong personal suspicion

**17) When do you think you got (or might have got) COVID-19?**
1)First time: DD/MM/YYYY
2) Second time: DD/MM/YYYY

**English Version of Data Collection Form-For Cases**

3)Third time: DD/MM/YYYY 4) Fourth time: DD/MM/YYYY

**Your medical history**

**18) In general, in the 3 months before the COVID-19 outbreak in March 2020, would you say your health was…**

1) Very good 2) Good 3) Fair 4) Poor

**For the item numbers 19 to 48, Which of the following symptoms did you have during the first 4 weeks of infection with COVID-19?** ***(Tick all that apply)***

| **BREATHING**: | 1 | 2 | 3 |
|---|---|---|---|
| **19)** Shortness of breath | None | Mild to moderate | Moderate to severe |
| **20)** Breath faster than usual or wake up at night due to shortness of breath | Never | Infrequently | Frequently |
| **PAIN**: | 1 | 2 | 3 |
| **21)** Chest pain, pain on breathing, stabbing or burning pain in any place on your body, aching all over the body, headache | None | Mild to moderate | Moderate to severe |
| **CIRCULATION:** | 1 | 2 | 3 |
| **22)** Palpitations, feel faint, dizziness on feet, swelling of extremities, your face, lips, tongue, throat, or cold extremities (which lasted more than usual or were colder than usual) | None | Mild to moderate | Moderate to severe |
| **FATIGUE:** | 1 | 2 | 3 |
| **23)** Tiredness, fatigue (feeling of physical or mental exhaustion that does not improve with rest) worsening of your symptoms following simple physical or mental activities | None | Mild to moderate | Moderate to severe |
| **MEMORY, THINKING AND COMMUNICATION:** | 1 | 2 | 3 |
| **24)** Brain fog (feeling sluggish, jet-lagged, or blanking out), confusion, memory loss, difficulty concentrating or planning, word-finding difficulties | None | Mild to moderate | Moderate to severe |
| **25)** Difficulty understanding what others were saying, slurred speech, reading difficulty (not related to dyslexia) | None | Mild to moderate | Moderate to severe |
| **MOVEMENT:** | 1 | 2 | 3 |
| **26)** Tremor, balance difficulty, difficulty with movement and coordination (uncontrollable shaking or trembling in part of your body) | None | Mild to moderate | Moderate to severe |

## English Version of Data Collection Form-For Cases

| | | | |
|---|---|---|---|
| **SLEEP:** | 1 | 2 | 3 |
| **27)** Problems falling asleep, sleep shorter or longer than usual, sleep interruption | Never | Infrequently | Frequently |
| **EARS-NOSE-THROAT:** | 1 | 2 | 3 |
| **28)** Earache, tinnitus, sensitivity to sounds, new hearing loss | None | Mild to moderate | Moderate to severe |
| **29)** Altered sense of smell or taste sneezing**,** runny nose**,** sinus congestion (discomfort or feeling of 'fullness' around nose, cheeks, forehead, or around the eyes) | None | Mild to moderate | Moderate to severe |
| **30)** Production of mucus, cough, sore throat, hoarse voice, difficulty swallowing, mouth ulcers, dry mouth, worsening of known dental problems | None | Mild to moderate | Moderate to severe |
| **STOMACH AND DIGESTION:** | 1 | 2 | 3 |
| **31)** Belly pain or bloating, nausea, indigestion, heartburn, diarrhea, constipation | None | Mild to moderate | Moderate to severe |
| **32)** Unplanned weight loss or weight gain | Never | Infrequently | Frequently |
| **MUSCLES AND JOINTS:** | 1 | 2 | 3 |
| **33)** Muscle pain or weakness or stiffness, muscle twitching or cramping, tingling and numbness | None | Mild to moderate | Moderate to severe |
| **34)** Joint pain or swelling or stiffness | None | Mild to moderate | Moderate to severe |
| **MENTAL HEALTH AND WELLBEING:** | 1 | 2 | 3 |
| **35)** Lack of interest, sadness, mood swings, feel lonely, change in appetite, not hopeful about the future, anxiety | None | Mild to moderate | Moderate to severe |
| **36)** Thoughts about harming yourself, feel like you are not the person you were before having COVID-19 | Never | Infrequently | Frequently |
| **SKIN AND HAIR:** | 1 | 2 | 3 |
| **37)** Dry or itchy skin | None | Mild to moderate | Moderate to severe |
| **38)** Purple-red spots on your feet, rashes, hives | None | Mild to moderate | Moderate to severe |
| **39)** Hair loss, changes to your nails (ridging, pitting, discoloration, or brittle nails) | None | Mild to moderate | Moderate to severe |

**English Version of Data Collection Form-For Cases**

| **Eyes:** | 1 | 2 | 3 |
|---|---|---|---|
| **40)** Red or dry or itchy or watery eyes, pressure behind your eyes, flashing lights , foreign body sensation | Never | Infrequently | Frequently |
| **41)** Pain behind your eyes, blurred vision, double vision (not related to wearing glasses), sensitivity to light | None | Mild to moderate | Moderate to severe |
| **REPRODUCTIVE AND SEXUAL HEALTH:**<br>, | 1 | 2 | 3 |
| **Female or male:**<br>**42)** **In female**: changes to your menstrual period, worsening premenstrual syndrome (PMS) have blood clots more than usual, worried about your ability to have an orgasm<br>**In male**: decreased interest in sex, difficulty with ejaculation | Never | Infrequently | Frequently |
| **Female or male:**<br>**43)** **In female**: vaginal dryness, discharge, decreased interest in sex<br>**In male**: keeping an erection | None | Mild to moderate | Moderate to severe |
| **UROLOGICAL SYMPTOMS:** | 1 | 2 | 3 |
| **44)** Loss of control of urine (leakage), difficulty passing urine, increased thirst or passing more urine than usual | Never | Infrequently | Frequently |
| **IMMUNOLOGICAL SYMPTOMS:** | 1 | 2 | 3 |
| **45)** Heightened reaction to known or new allergies | Never | Infrequently | Frequently |
| **OTHERS:** | 1 | 2 | 3 |
| **46)** Fever or chills | Never | Infrequently | Frequently |
| **47)** Sweating problem, hot flushes, the swelling of your glands (lymph nodes), or your vertigo (when everything around you was<br>spinning enough to affect your balance) | None | Mild to moderate | Moderate to severe |
| **IMPACT ON DAILY LIFE** | 1 | 2 | 3 |
| **48)** Your symptoms affected your ability to work, volunteer, go to school or take part in organized activities, go shopping, do housework or light chores, move around easily, look after yourself, relationships with friends and family, socialize and interact with others, or enjoy life | Not at all | Moderately | Severely |

**49) In the first 4 weeks of your illness, did you look for any medical help for any symptoms you think may have been caused by COVID-19? *(Tick all that apply)*.**

1)No

2)Yes – discussed symptoms with doctor/GP

3)Yes – accessed online advice at emergency medical services (115)

4)Yes – visited the pharmacy to receive medication without prescription

**50) Have you ever had to stay in hospital because of COVID-19 symptoms?**
1)No 2) Yes, less than 2 weeks since COVID-19 infection

3)Yes, 2-4 weeks since COVID-19 infection 4) Yes, more than 1 month since COVID-19 infection

**51) Did you look for any medical help for any symptoms you had more than 4 weeks after your symptoms of COVID-19 began?** ***(Tick all that apply)***
1)No

2)Yes – discussed symptoms with doctor/GP

3)Yes – accessed online advice at emergency medical services (115)

4)Yes – visited the pharmacy to receive medication without prescription

**52) How long have you had / did you have COVID-19 symptoms after 4 weeks of initiation of the <u>first</u> infection with COVID-19?**
1)1-2 weeks 2)3-4 weeks 3)1-3 months 4) more than 3 months

**53) How long have you had / did you have COVID-19 symptoms after 4 weeks of initiation of the <u>second</u> infection with COVID-19?**
1)1-2 weeks 2)3-4 weeks 3)1-3 months 4) more than 3 months

**54) How long have you had / did you have COVID-19 symptoms after 4 weeks of initiation of the <u>third</u> infection with COVID-19?**
1)1-2 weeks 2)3-4 weeks 3)1-3 months 4) more than 3 months

**55) What help or support have you found helpful, 4 weeks after initiation of your COVID-19 illness?**
1)None

2)Support from family or people you live with

3)Support from neighbors or friends

4)Su pport from a religious group or charity or Self-organized group or network of people with the same condition, e.g. on social media

**Vaccination**

**56) Have you had COVID-19 vaccine injection?**
1)No, I did not receive any 2) Yes, only one dose 3) Yes, two doses 4) Yes, three or more doses

**57) When was your COVID-19 vaccine injection? If you can't remember exactly, please put your best estimate.** ***(Write all that apply)***

1)First dose: (DD / MM / YYYY) _ _ / _ _ / _ _ _ _ 2) Second dose: (DD / MM / YYYY) _ _ / _ _ / _ _ _ _

3) Third dose: (DD / MM / YYYY) _ _ / _ _ / _ _ _ _ 4) Fourth dose: (DD / MM / YYYY) _ _ / _ _ / _ _ _ _

**58) When did you be infected with COVID-19 after your COVID-19 vaccine injection?** ***(write all that apply)***

1)Never 2) After the first dose

3)After the second dose 4) After the third or more doses

**59) For how long were you unable to function as normal due to COVID-19 symptoms?**

1)I was always able to function as normal 2)1-2 weeks

3)3 weeks to 1 month 4) More than 1 month

**In items 60-89 we would like to know if you have had ANY of the following symptoms after 1 month from COVID-19 infection or other respiratory infection.**

| **BREATHING**: | 1 | 2 | 3 |
|---|---|---|---|
| **60)** Shortness of breath | None | Mild to moderate | Moderate to severe |
| **61)** Breathe faster than usual **or** wake up at night due to shortness of breath | None | Mild to moderate | Moderate to severe |
| **PAIN**: | 1 | 2 | 3 |
| **62)** Chest pain, pain on breathing, stabbing or burning pain in any place on your body, aching all over the body, headache | None | Mild to moderate | Moderate to severe |
| **CIRCULATION:** | 1 | 2 | 3 |
| **63)** Palpitations, feel faint, dizziness on feet, swelling of extremities, your face, lips, tongue, throat, or cold extremities (which lasted more than usual or were colder than usual) | None | Mild to moderate | Moderate to severe |
| **FATIGUE:** | 1 | 2 | 3 |

**English Version of Data Collection Form-For Cases**

| | | | |
|---|---|---|---|
| **64)** Tiredness, fatigue (feeling of physical or mental exhaustion that does not improve with rest)<br>worsening of your symptoms following simple physical or mental activities | None | Mild to moderate | Moderate to severe |
| **MEMORY, THINKING AND COMMUNICATION:** | 1 | 2 | 3 |
| **65)** Brain fog (feeling sluggish, jet-lagged, or blanking out) ,confusion, memory loss, difficulty concentrating or planning, word-finding difficulties | None | Mild to moderate | Moderate to severe |
| **66)** Difficulty understanding what others were saying, slurred speech, reading difficulty (not related to dyslexia) | None | Mild to moderate | Moderate to severe |
| **MOVEMENT:** | 1 | 2 | 3 |
| **67)** Tremor, balance difficulty, difficulty with movement and coordination (uncontrollable shaking or trembling in part of your body) | None | Mild to moderate | Moderate to severe |
| **SLEEP:** | 1 | 2 | 3 |
| **68)** Problems falling asleep, sleep shorter or longer than usual, sleep interruption | Never | Infrequently | Frequently |
| **EARS-NOSE-THROAT:** | 1 | 2 | 3 |
| **69)** Earache, tinnitus, sensitivity to sounds, new hearing loss | None | Mild to moderate | Moderate to severe |
| **70)** Altered sense of smell or taste sneezing**,** runny nose**,** sinus congestion (discomfort or feeling of 'fullness' around nose, cheeks, forehead, or around the eyes) | None | Mild to moderate | Moderate to severe |
| **71)** Production of mucus, cough, sore throat, hoarse voice, difficulty swallowing, mouth ulcers, dry mouth ,worsening of known dental problems | None | Mild to moderate | Moderate to severe |
| **STOMACH AND DIGESTION:** | 1 | 2 | 3 |
| **72)** Belly pain or bloating, nausea, indigestion, heartburn, diarrhea, constipation | None | Mild to moderate | Moderate to severe |
| **73)** Unplanned weight loss or weight gain | Never | Infrequently | Frequently |
| **MUSCLES AND JOINTS:** | 1 | 2 | 3 |
| **74)** Muscle pain or weakness or stiffness, muscle twitching or cramping, tingling and numbness | None | Mild to moderate | Moderate to severe |
| **75)** Joint pain or swelling or stiffness | None | Mild to moderate | Moderate to severe |
| **MENTAL HEALTH AND WELLBEING:** | 1 | 2 | 3 |

## English Version of Data Collection Form-For Cases

| | | | |
|---|---|---|---|
| **76)** Lack of interest, sadness, mood swings, feel lonely, change in appetite, not hopeful about the future, anxiety | None | Mild to moderate | Moderate to severe |
| **77)** Thoughts about harming yourself, feel like you are not the person you were before having COVID-19 | Never | Infrequently | Frequently |
| **SKIN AND HAIR:** | 1 | 2 | 3 |
| **78)** Dry or itchy skin | None | Mild to moderate | Moderate to severe |
| **79)** Purple-red spots on your feet, rashes, hives | Never | Infrequently | Frequently |
| **80)** Hair loss, changes to your nails (ridging, pitting, discoloration, or brittle nails) | None | Mild to moderate | Moderate to severe |
| **Eyes:** | 1 | 2 | 3 |
| **81)** Red or dry or itchy or watery eyes, pressure behind your eyes, flashing lights, foreign body sensation | Never | Infrequently | Frequently |
| **82)** Pain behind your eyes, blurred vision, double vision (not related to wearing glasses), sensitivity to light | None | Mild to moderate | Moderate to severe |
| **REPRODUCTIVE AND SEXUAL HEALTH:** | 1 | 2 | 3 |
| **Female or male:**<br>**83)** **In female**: changes to your menstrual period, worsening premenstrual syndrome (PMS) have blood clots more than usual, worried about your ability to have an orgasm<br>**In male**: decreased interest in sex, difficulty with ejaculation | Never | Infrequently | Frequently |
| **Female or male:**<br>**84)** **In female**: vaginal dryness, discharge, decreased interest in sex<br>**In male**: keeping an erection | None | Mild to moderate | Moderate to severe |
| **UROLOGICAL SYMPTOMS:** | 1 | 2 | 3 |
| **85)** Loss of control of urine (leakage), difficulty passing urine, increased thirst or passing more urine than usual | Never | Infrequently | Frequently |
| **IMMUNOLOGICAL SYMPTOMS:** | 1 | 2 | 3 |
| **86)** Heightened reaction to known or new allergies | Never | Infrequently | Frequently |
| **OTHERS:** | 1 | 2 | 3 |
| **87)** Fever or chills | Never | Infrequently | Frequently |
| **88)** Sweating problem, hot flushes, the swelling of your glands (lymph nodes), or your vertigo (when everything around you was spinning enough to affect your balance) | None | Mild to moderate | Moderate to severe |
| **IMPACT ON DAILY LIFE** | 1 | 2 | 3 |

| | | | |
|---|---|---|---|
| **89)** Your symptoms affected your ability to work, volunteer, go to school or take part in organized activities, go shopping, do housework or light chores, move around easily, look after yourself, relationships with friends and family, socialize and interact with others, or enjoy life | Not at all | Moderately | Severely |

**90) At present time, I think that my health status is as good as before the COVID-19 outbreak in March 2020.**

1) Not at all   2) Very little   3) To Some extent   4) Completely

**English Version of Data Collection Form-For Controls**

**NON-CASES (GROUP 2)**

**Personal information:**

2) **Age**: ……. / 2) **Sex**: Male☐[1] Female☐[2] / 3) **Weight**: ……. / 4) **Height**: …….

5) **Level of Education**: Illiterate ☐[1] Less than 6th grade ☐[2] Less than high school diploma ☐[3] high school diploma ☐[4] Technician degree ☐[5] Bachelor or master degree ☐[6] Doctorate degree ☐[7]

6) **Employment status**: Employed☐[1] Unemployed☐[2] Housewife☐[3] Post-High School student☐[4] Retired☐[5]

7) **Marital status**: Single☐[1] Married☐[2] Divorced☐[3] Widow☐[4]

**Did you already have any of the following health problems or conditions since before COVID-19 pandemic or before being infected with COVID-19?** ***(Tick all that apply)***

| | 1 | 2 |
|---|---|---|
| **8)** Lung diseases | No | Yes |
| **9)** Diabetes | No | Yes |
| **10)** Cardiovascular diseases (ex: heart failure, kidney diseases, hypertension) | No | Yes |
| **11)** Autoimmune diseases (ex: SLE, rheumatoid arthritis) | No | Yes |
| **12)** Malignancy (any type) | No | Yes |
| **13)** Medication (corticosteroids, chemotherapy) | No | Yes |

**14) What is your Smoking status?**

1)Non-Smoker

2) Ex-Smoker (stopped before COVID-19 outbreak)

3)Ex-Smoker (stopped after COVID-19 outbreak)

4) Current smoker

**************During data entry, three numbers should be kept empty*********************

**Your medical history**

**18) In general, in the 3 months before the COVID-19 outbreak in March 2020, would you say your health was…**

2) Very good 2) Good 3) Fair 4) Poor

**For the item numbers 19 to 47, Which of the following symptoms did you already have if you have not infected with COVID-19?** ***(Tick all that apply)***

## English Version of Data Collection Form-For Controls

| | | | |
|---|---|---|---|
| **BREATHING**: | 1 | 2 | 3 |
| **19)** Shortness of breath | None | Mild to moderate | Moderate to severe |
| **20)** Breath faster than usual or wake up at night due to shortness of breath | Never | Infrequently | Frequently |
| **PAIN**: | 1 | 2 | 3 |
| **21)** Chest pain, pain on breathing, stabbing or burning pain in any place on your body, aching all over the body, headache | None | Mild to moderate | Moderate to severe |
| **CIRCULATION:** | 1 | 2 | 3 |
| **22)** Palpitations, feel faint, dizziness on feet, swelling of extremities, your face, lips, tongue, throat, or cold extremities (which lasted more than usual or were colder than usual) | None | Mild to moderate | Moderate to severe |
| **FATIGUE:** | 1 | 2 | 3 |
| **23)** Tiredness, fatigue (feeling of physical or mental exhaustion that does not improve with rest)<br>worsening of your symptoms following simple physical or mental activities | None | Mild to moderate | Moderate to severe |
| **MEMORY, THINKING AND COMMUNICATION:** | 1 | 2 | 3 |
| **24)** Brain fog (feeling sluggish, jet-lagged, or blanking out), confusion, memory loss, difficulty concentrating or planning, word-finding difficulties | None | Mild to moderate | Moderate to severe |
| **25)** Difficulty understanding what others were saying, slurred speech, reading difficulty (not related to dyslexia) | None | Mild to moderate | Moderate to severe |
| **MOVEMENT:** | 1 | 2 | 3 |
| **26)** Tremor, balance difficulty, difficulty with movement and coordination (uncontrollable shaking or trembling in part of your body) | None | Mild to moderate | Moderate to severe |
| **SLEEP:** | 1 | 2 | 3 |
| **27)** Problems falling asleep, sleep shorter or longer than usual, sleep interruption | Never | Infrequently | Frequently |
| **EARS-NOSE-THROAT:** | 1 | 2 | 3 |
| **28)** Earache, tinnitus, sensitivity to sounds, new hearing loss | None | Mild to moderate | Moderate to severe |

**English Version of Data Collection Form-For Controls**

| | | | |
|---|---|---|---|
| **29)** Altered sense of smell or taste sneezing**,** runny nose**,** sinus congestion (discomfort or feeling of 'fullness' around nose, cheeks, forehead, or around the eyes) | None | Mild to moderate | Moderate to severe |
| **30)** Production of mucus, cough, sore throat, hoarse voice, difficulty swallowing, mouth ulcers, dry mouth, worsening of known dental problems | None | Mild to moderate | Moderate to severe |
| **STOMACH AND DIGESTION:** | 1 | 2 | 3 |
| **31)** Belly pain or bloating, nausea, indigestion, heartburn, diarrhea, constipation | None | Mild to moderate | Moderate to severe |
| **32)** Unplanned weight loss or weight gain | Never | Infrequently | Frequently |
| **MUSCLES AND JOINTS:** | 1 | 2 | 3 |
| **33)** Muscle pain or weakness or stiffness, muscle twitching or cramping, tingling and numbness | None | Mild to moderate | Moderate to severe |
| **34)** Joint pain or swelling or stiffness | None | Mild to moderate | Moderate to severe |
| **MENTAL HEALTH AND WELLBEING:** | 1 | 2 | 3 |
| **35)** Lack of interest, sadness, mood swings, feel lonely, change in appetite, not hopeful about the future, anxiety | None | Mild to moderate | Moderate to severe |
| **36)** Thoughts about harming yourself, feel like you are not the person you were before having COVID-19 | Never | Infrequently | Frequently |
| **SKIN AND HAIR:** | 1 | 2 | 3 |
| **37)** Dry or itchy skin | None | Mild to moderate | Moderate to severe |
| **38)** Purple-red spots on your feet, rashes, hives | None | Mild to moderate | Moderate to severe |
| **39)** Hair loss, changes to your nails (ridging, pitting, discoloration, or brittle nails) | None | Mild to moderate | Moderate to severe |
| **Eyes:** | 1 | 2 | 3 |
| **40)** Red or dry or itchy or watery eyes, pressure behind your eyes, flashing lights , foreign body sensation | Never | Infrequently | Frequently |
| **41)** Pain behind your eyes, blurred vision, double vision (not related to wearing glasses), sensitivity to light | None | Mild to moderate | Moderate to severe |
| **REPRODUCTIVE AND SEXUAL HEALTH:** , | 1 | 2 | 3 |

**English Version of Data Collection Form-For Controls**

| | | | |
|---|---|---|---|
| **Female or male:**<br>**42) In female**: changes to your menstrual period, worsening premenstrual syndrome (PMS) have blood clots more than usual, worried about your ability to have an orgasm<br>**In male**: decreased interest in sex, difficulty with ejaculation | Never | Infrequently | Frequently |
| **Female or male:**<br>**43) In female**: vaginal dryness, discharge, decreased interest in sex<br>**In male**: keeping an erection | None | Mild to moderate | Moderate to severe |
| **UROLOGICAL SYMPTOMS:** | 1 | 2 | 3 |
| **44)** Loss of control of urine (leakage), difficulty passing urine, increased thirst or passing more urine than usual | Never | Infrequently | Frequently |
| **IMMUNOLOGICAL SYMPTOMS:** | 1 | 2 | 3 |
| **45)** Heightened reaction to known or new allergies | Never | Infrequently | Frequently |
| **OTHERS:** | 1 | 2 | 3 |
| **46)** Fever or chills | Never | Infrequently | Frequently |
| **47)** Sweating problem, hot flushes, the swelling of your glands (lymph nodes), or your vertigo (when everything around you was spinning enough to affect your balance) | None | Mild to moderate | Moderate to severe |

**************During data entry, eight numbers should be kept empty*******************

## Vaccination

**56) Have you had COVID-19 vaccine injection?**

1)No, I did not receive any 2) Yes, only one dose 3) Yes, two doses 4) Yes, three or more doses

**57) When was your COVID-19 vaccine injection? If you can't remember exactly, please put your best estimate.** ***(Write all that apply)***

1)First dose: (DD / MM / YYYY) _ _ / _ _ / _ _ _ _ 2) Second dose: (DD / MM / YYYY) _ _ / _ _ / _ _ _ _

3) Third dose: (DD / MM / YYYY) _ _ / _ _ / _ _ _ _   4) Fourth dose: (DD / MM / YYYY) _ _ / _ _ / _ _ _ _

**************During data entry, thirty-two numbers should be kept empty*****************

**90) At present time, I think that my health status is as good as before the COVID-19 outbreak in March 2020.**

2) Not at all   2) Very little   3) To Some extent   4) Completely

**Persian Version of Data Collection Form- For Cases**

## گروه با سابقه ابتلا به کووید-۱۹

**مقایسه شیوع علائم کووید-۱۹ طولانی در بین جمعیت بزرگسال با و بدون سابقه ابتلا به کووید-۱۹ در استان فارس، ۱۳۹۸-۱۴۰۲**

**اطلاعات شخصی:**

**۱) سن:** ....... / **۲) جنسیت:** مرد☐[۱] زن☐[۲] / **۳) وزن (کیلوگرم) :** ....... / **۴) قد (سانتی متر) :** .......

**۵) سطح تحصیلات:** بی سواد ☐[۱] کمتر از ششم ☐[۲] ششم تا یازدهم ☐[۳] دیپلم دبیرستان ☐[۴] فوق دیپلم☐[۵] لیسانس☐[۶] فوق لیسانس ☐[۷] مدرک دکترا ☐[۸]

**۶) وضعیت اشتغال:** شاغل☐[۱] بیکار☐[۲] خانه دار☐[۳] دانشجو☐[۴] بازنشسته☐[۵]

**۷) وضعیت تاهل:** مجرد☐[۱] متاهل☐[۲] مطلقه☐[۳] بیوه☐[۴]

**کدامیک از موارد زیر قبل از همه گیری کرونا (یا قبل از ابتلا به کرونا) در مورد شما وجود داشته است؟ (همه موارد را پاسخ دهید.)**

| | ۱ | ۲ |
|---|---|---|
| **۸)** آسم یا سایر بیماری های ریوی | بله[۱] | خیر[۲] |
| **۹)** دیابت | بله[۱] | خیر[۲] |
| **۱۰)** بیماری های قلب و عروق (مانند نارسایی قلب، بیماری های کلیوی، فشار خون بالا) | بله[۱] | خیر[۲] |
| **۱۱)** بیماری های خود ایمنی (مانند لوپوس، روماتیسم) | بله[۱] | خیر[۲] |
| **۱۲)** سرطان (هر نوع) | بله[۱] | خیر[۲] |
| **۱۳)** مصرف دارو (کورتون ها مانند پردنیزولون، داروهای شیمی درمانی) | بله[۱] | خیر[۲] |

**۱۴) وضعیت سیگار کشیدن شما چگونه است؟**

۱) غیر سیگاری (مساوی یا کمتر از ۱۰۰ نخ سیگار در طول عمر)

۲) سیگاری سابق (بیشتر از ۱۰۰ نخ سیگار در طول عمر اما ترک قبل از شیوع کرونا)

۳) سیگاری سابق (بیشتر از ۱۰۰ نخ سیگار در طول عمر اما ترک پس از شیوع کرونا و حداقل یک ماه است که سیگار نمیکشم )

۴) سیگاری فعلی (بیشتر از ۱۰۰ نخ سیگار در طول عمر و مصرف کننده فعلی)

**۱۵) آیا فکر می کنید که در حال حاضر مبتلا به کرونا هستید یا تا به حال مبتلا شده اید؟**

۱) خیر　　　۲) بله، با تست مثبت (PCR (آزمایش خون) ، تست سریع) تایید شده است.

۳) بله، بر اساس نظر پزشک　　　۴) بله، بر اساس گمان خود یا خانواده ام

**۱۶) آیا فکر می کنید بیش از یک بار به کرونا مبتلا شده اید؟**

۱) خیر　　　۲) بله، با تست مثبت (PCR (آزمایش خون) ، تست سریع) تایید شده است.

۳) بله، بر اساس نظر پزشک　　　۴) بله، بر اساس گمان خود یا خانواده ام

**۱۷) فکر می کنید در چه تاریخی به کرونا مبتلا شده اید؟ (یا ممکن است به آن مبتلا شده باشید؟)**

۱) بار اول: -- / -- / ----　　۲) بار دوم: -- / -- / ----　　۳) بار سوم: -- / -- / ----　　۴) بار چهارم: -- / -- / ----

**سابقه پزشکی شما**

**۱۸) وضعیت سلامت شما به طور کلی تا ۳ ماه قبل از شیوع کرونا در اسفند ۱۳۹۸، چگونه بود؟**

۱) خیلی خوب　　۲) خوب　　۳) متوسط　　۴) ضعیف

## Persian Version of Data Collection Form- For Cases

**در طول ۴ هفته اول ابتلا به کرونا ، کدام یک از علائم مربوط به سوالات ۱۹ تا ۴۸ را داشتید ؟**

| | | | |
|---|---|---|---|
| **تنفس :** | **۱** | **۲** | **۳** |
| **۱۹)** تنگی نفس | نداشتم[۱] | خفیف تا متوسط[۲] | متوسط تا شدید[۳] |
| **۲۰)** تنفس سریعتر از حد معمول یا بیدار شدن از خواب به دلیل تنگی نفس | هرگز[۱] | گاهی اوقات[۲] | غالب اوقات[۳] |
| **درد :** | **۱** | **۲** | **۳** |
| **۲۱)** درد قفسه سینه، درد در هنگام تنفس، درد در هر نقطه از بدن، درد در تمام بدن ، سر درد | نداشتم[۱] | خفیف تا متوسط[۲] | متوسط تا شدید[۳] |
| **گردش خون :** | **۱** | **۲** | **۳** |
| **۲۲)** تپش قلب، احساس ضعف، احساس سرگیجه حین ایستادن، ورم اندام ها یا صورت یا لب ها یا زبان یا گلو ، اندام های سرد (که بیشتر از حد معمول طول بکشد یا سرد تر از حد معمول باشد) هیچ خفیف متوسط شدید | نداشتم[۱] | خفیف تا متوسط[۲] | متوسط تا شدید[۳] |
| **خستگی :** | ۱ | ۲ | ۳ |
| **۲۳)** خستگی شدید ، احساس خستگی جسمی یا روحی که با استراحت بهبود نمی یابد ، بدتر شدن علائم شما به دنبال فعالیت های فیزیکی یا ذهنی ساده | نداشتم[۱] | خفیف تا متوسط[۲] | متوسط تا شدید[۳] |
| **حافظه، تفکر و ارتباط:** | **۱** | **۲** | **۳** |
| **۲۴)** مه مغزی (احساس کندی ، خالی شدن فکر)، گیجی، مشکلات حافظه ، مشکل در تمرکز یا برنامه ریزی، مشکلات کلمه یابی حین صحبت کردن | نداشتم[۱] | خفیف تا متوسط[۲] | متوسط تا شدید[۳] |
| **۲۵)** مشکل در درک آنچه دیگران می گویند، گفتار نامفهوم، مشکل در خواندن | نداشتم[۱] | خفیف تا متوسط[۲] | متوسط تا شدید[۳] |
| **حرکت:** | **۱** | **۲** | **۳** |
| **۲۶)** لرزش، مشکل تعادل، مشکل در حرکت و هماهنگی حرکات (لرزش غیرقابل کنترل در بخشی از بدن شما) | نداشتم[۱] | خفیف تا متوسط[۲] | متوسط تا شدید[۳] |
| **خواب:** | **۱** | **۲** | **۳** |
| **۲۷)** مشکلات به خواب رفتن، خواب کوتاهتر یا طولانی تر از حد معمول، وقفه در خواب | هرگز[۱] | گاهی اوقات[۲] | غالب اوقات[۳] |
| **گوش، حلق و بینی:** | **۱** | **۲** | **۳** |
| **۲۸)** گوش درد، وزوز گوش، حساسیت غیر عادی به صداها، کم شنوایی که اخیرا ایجاد شده باشد | نداشتم[۱] | خفیف تا متوسط[۲] | متوسط تا شدید[۳] |

# Persian Version of Data Collection Form- For Cases

| | | | |
|---|---|---|---|
| **۲۹)** تغییر در حس بویایی یا چشایی، عطسه، آبریزش بینی، احتقان در سینوس ها (ناراحتی یا احساس «پری» در اطراف بینی، گونه ها، پیشانی یا اطراف چشم) | نداشتم[۱] | خفیف تا متوسط[۲] | متوسط تا شدید[۳] |
| **۳۰)** تولید خلط ، سرفه، گلودرد، صدای خشن، اشکال در بلع، زخم های دهان، خشکی دهان، بدتر شدن مشکلات دندانی | نداشتم[۱] | خفیف تا متوسط[۲] | متوسط تا شدید[۳] |
| **معده و گوارش:** | **۱** | **۲** | **۳** |
| **۳۱)** شکم درد یا نفخ، حالت تهوع، سوء هاضمه، سوزش سر دل، اسهال، یبوست | نداشتم[۱] | خفیف تا متوسط[۲] | متوسط تا شدید[۳] |
| **۳۲)** کاهش یا افزایش وزن بدون برنامه ریزی | هرگز[۱] | گاهی اوقات[۲] | غالب اوقات[۳] |
| **ماهیچه ها و مفاصل:** | ۱ | ۲ | ۳ |
| **۳۳)** درد یا ضعف یا سفتی عضلات، انقباض یا گرفتگی عضلات، گزگز و بی حسی | نداشتم[۱] | خفیف تا متوسط[۲] | متوسط تا شدید[۳] |
| **۳۴)** درد یا تورم یا سفتی مفاصل | نداشتم[۱] | خفیف تا متوسط[۲] | متوسط تا شدید[۳] |
| **سلامت روان و رفاه:** | **۱** | **۲** | **۳** |
| **۳۵)** لذت نبردن ، غمگینی، نوسانات خلقی، احساس تنهایی، تغییر در اشتها، ناامیدی نسبت به آینده، اضطراب | نداشتم[۱] | خفیف تا متوسط[۲] | متوسط تا شدید[۳] |
| **۳۶)** داشتن افکاری در مورد آسیب رساندن به خود یا احساس تغییر شخصیت نسبت به قبل از ابتلا به کرونا | هرگز[۱] | به ندرت[۲] | گاهی[۳] |
| **پوست و مو:** | **۱** | **۲** | **۳** |
| **۳۷)** خشکی یا خارش پوست | نداشتم[۱] | خفیف تا متوسط[۲] | متوسط تا شدید[۳] |
| **۳۸)** لکه های بنفش قرمز روی پاها، ضایعات پوستی، کهیر | هرگز[۱] | گاهی اوقات[۲] | غالب اوقات[۳] |
| **۳۹)** ریزش مو، تغییر در ناخن ها (ایجاد خطوط عمودی روی ناخن ، ایجاد حفره روی ناخن، تغییر رنگ یا شکنندگی ناخن ها) | نداشتم[۱] | خفیف تا متوسط[۲] | متوسط تا شدید[۳] |
| **چشم:** | **۱** | **۲** | **۳** |
| **۴۰)** قرمزی یا خشکی یا خارش یا آبریزش چشم، فشار پشت چشم، دیدن نور چشمک زن، احساس جسم خارجی در چشم | هرگز[۱] | گاهی اوقات[۲] | غالب اوقات[۳] |
| **۴۱)** درد پشت چشم، تاری دید، دوبینی، حساسیت به نور | نداشتم[۱] | خفیف تا متوسط[۲] | متوسط تا شدید[۳] |

| سلامت باروری و جنسی: | ۱ | ۲ | ۳ |
|---|---|---|---|
| **زن یا مرد:**<br>**۴۲) در پاسخ دهنده ی زن:** تغییرات در دوره قاعدگی، بدتر شدن علایم پیش از قاعدگی، لخته های خونی بیش از حد معمول در دوران قاعدگی<br>**در پاسخ دهنده ی مرد:** کاهش علاقه به رابطه جنسی، مشکل در انزال، نگرانی در مورد توانایی ارضای جنسی | هرگز[۱] | گاهی اوقات[۲] | غالب اوقات[۳] |
| **زن یا مرد:**<br>**۴۳) در پاسخ دهنده ی زن:** خشکی واژن، ترشح، کاهش علاقه به رابطه جنسی<br>**در پاسخ دهنده ی مرد:** حفظ نعوظ، کاهش علاقه به رابطه جنسی | نداشتم[۱] | خفیف تا متوسط[۲] | متوسط تا شدید[۳] |
| **علائم ادراری:** | **۱** | **۲** | **۳** |
| **۴۴)** از دست دادن کنترل ادرار (نشت)، مشکل در دفع ادرار، دفع ادرار بیشتر از حد معمول | هرگز[۱] | گاهی اوقات[۲] | غالب اوقات[۳] |
| **علائم مربوط به سیستم ایمنی:** | **۱** | **۲** | **۳** |
| **۴۵)** آلرژی شدید (از قبل یا جدید) | هرگز[۱] | گاهی اوقات[۲] | غالب اوقات[۳] |
| **سایر موارد:** | **۱** | **۲** | **۳** |
| **۴۶)** تب یا لرز | هرگز[۱] | گاهی اوقات[۲] | غالب اوقات[۳] |
| **۴۷)** زیاد عرق کردن، گرگرفتگی، تورم غدد لنفاوی، سرگیجه (زمانی که همه چیز در اطراف شما بچرخد به نحوی که تعادل شما را تحت تاثیر قرار دهد) | نداشتم[۱] | خفیف تا متوسط[۲] | متوسط تا شدید[۳] |
| **تأثیر بر زندگی روزانه:** | **۱** | **۲** | **۳** |
| **۴۸)** تغییر در توانایی شما برای انجام کار، رفتن به مدرسه، خرید، انجام کارهای خانه یا کارهای سبک، حرکات آسان، مراقبت از خود، روابط با دوستان و خانواده، لذت بردن از زندگی | اصلا[۱] | تا حدودی[۲] | قابل توجه[۳] |

**۴۹) در یک ماه اول ابتلا به کرونا ، برای علائمی که فکر می کردید ناشی از کرونا است ، به دنبال کمک پزشکی بودید؟ (میتوانید بیش از یک مورد را علامت بزنید).**

۱) خیر

۲) بله - صحبت با پزشک در مورد علائم یا مراجعه به مراکز بهداشتی درمانی

۳) بله – مشاوره آنلاین مثلا اورژانس ۱۱۵

۴) بله - مراجعه به داروخانه برای دریافت دارو بدون نسخه

**۵۰) آیا تا به حال به دلیل بیماری کرونا در بیمارستان بستری شده اید؟**

۱) خیر ۲) بله، کمتر از ۲ هفته بعد از ابتلا به کرونا

۳) بله، ۲ هفته تا یک ماه بعد از ابتلا به کرونا ۴) بله، بیش از ۱ ماه بعد از ابتلا به کرونا

**۵۱) آیا برای علائمی که بیش از یک ماه پس از ابتلا به کرونا داشتید به دنبال کمک پزشکی بودید؟ (میتوانید بیش از یک مورد را علامت بزنید).**

۱) خیر

۲) بله - صحبت با پزشک در مورد علائم یا مراجعه به مراکز بهداشتی درمانی

۳) بله – مشاوره آنلاین مثلا اورژانس ۱۱۵

۴) بله - مراجعه به داروخانه برای دریافت دارو بدون نسخه

**۵۲) پس از گذشت یک ماه از اولین مرتبه ابتلا به کرونا ، تا چه مدت علایم کرونا را داشته اید؟**

۱) ۱-۲ هفته ۲) ۳ هفته تا یک ماه ۳) ۱-۳ ماه ۴) بیش از ۳ ماه

**۵۳) پس از گذشت یک ماه از دومین مرتبه ابتلا به کرونا ، تا چه مدت علایم کرونا را داشته اید؟**

۱) ۱-۲ هفته ۲) ۳ هفته تا یک ماه ۳) ۱-۳ ماه ۴) بیش از ۳ ماه

**۵۴) پس از گذشت یک ماه از سومین مرتبه ابتلا به کرونا ، تا چه مدت علایم کرونا را داشته اید؟**

۱) ۱-۲ هفته ۲) ۳ هفته تا یک ماه ۳) ۱-۳ ماه ۴) بیش از ۳ ماه

**۵۵) یک ماه پس از ابتلا به کرونا چه کمک یا حمایتی دریافت کردید؟ (میتوانید بیش از یک مورد را علامت بزنید).**

۱) هیچ حمایتی دریافت نکردم

۲) حمایت خانواده یا فرد/افرادی که با آنها زندگی می کنید

۳) حمایت همسایگان یا دوستان

۴) حمایت یک گروه مذهبی، خیریه، دولتی یا شبکه ای از افراد مبتلا به بیماری کرونا ( به عنوان مثال در رسانه های اجتماعی)

**واکسیناسیون**

**۵۶) آیا واکسن کرونا را تزریق کرده اید؟**

۱) خیر، من هیچ دوزی دریافت نکردم ۲) بله، فقط یک دوز ۳) بله، دو دوز ۴) بله، سه دوز یا بیشتر

**۵۷) در چه تاریخی واکسن کرونا را تزریق کردید ؟ اگر دقیقاً به خاطر نمی آورید، لطفاً بهترین تخمین خود را ارائه دهید. (میتوانید بیش از یک مورد را علامت بزنید).**

۱) بار اول: -- / -- / ---- ۲) بار دوم: -- / -- / ----

۳) بار سوم: -- / -- / ---- ۴) بار چهارم: -- / -- / ----

**۵۸) چه زمانی پس از تزریق واکسن کرونا به کرونا مبتلا شدید؟ (میتوانید بیش از یک مورد را علامت بزنید).**

۱) هرگز ۲) بعد از اولین دوز

۳) بعد از دوز دوم ۴) بعد از دوز سوم یا بیشتر

**۵۹) برای چه مدت به دلیل علائم کرونا عملکرد روزانه و معمول خود را از دست دادید؟**

۱) من همیشه عملکردی طبیعی داشتم. ۲) ۱-۲ هفته ۳) ۳ هفته تا یک ماه ۴) بیش از یک ماه

# Persian Version of Data Collection Form- For Cases

**در سؤال های۶۰-۸۹ میخواهیم بدانیم که پس از گذشت ۱ ماه از ابتلا به کرونا، کدامیک از علائم زیر را داشتید؟ (به تمامی سوالات پاسخ دهید.)**

| | | | |
|---|---|---|---|
| **تنفس :** | **۱** | **۲** | **۳** |
| **۶۰)** تنگی نفس | نداشتم[۱] | خفیف تا متوسط[۲] | متوسط تا شدید[۳] |
| **۶۱)** تنفس سریعتر از حد معمول یا بیدار شدن از خواب به دلیل تنگی نفس | هرگز[۱] | گاهی اوقات[۲] | غالب اوقات[۳] |
| **درد :** | **۱** | **۲** | **۳** |
| **۶۲)** درد قفسه سینه، درد در هنگام تنفس، درد در هر نقطه از بدن، درد در تمام بدن ، سر درد | نداشتم[۱] | خفیف تا متوسط[۲] | متوسط تا شدید[۳] |
| **گردش خون :** | **۱** | **۲** | **۳** |
| **۶۳)** تپش قلب، احساس ضعف، احساس سرگیجه حین ایستادن، ورم اندام ها یا صورت یا لب ها یا زبان یا گلو ، اندام های سرد (که بیشتر از حد معمول طول بکشد یا سرد تر از حد معمول باشد) هیچ خفیف متوسط شدید | نداشتم[۱] | خفیف تا متوسط[۲] | متوسط تا شدید[۳] |
| **خستگی :** | ۱ | ۲ | ۳ |
| **۶۴)** خستگی شدید ، احساس خستگی جسمی یا روحی که با استراحت بهبود نمی یابد ، بدتر شدن علائم شما به دنبال فعالیت های فیزیکی یا ذهنی ساده | نداشتم[۱] | خفیف تا متوسط[۲] | متوسط تا شدید[۳] |
| **حافظه، تفکر و ارتباط:** | **۱** | **۲** | **۳** |
| **۶۵)** مه مغزی (احساس کندی ، خالی شدن فکر)، گیجی، مشکلات حافظه ، مشکل در تمرکز یا برنامه ریزی، مشکلات کلمه یابی حین صحبت کردن | نداشتم[۱] | خفیف تا متوسط[۲] | متوسط تا شدید[۳] |
| **۶۶)** مشکل در درک آنچه دیگران می گویند، گفتار نامفهوم، مشکل در خواندن | نداشتم[۱] | خفیف تا متوسط[۲] | متوسط تا شدید[۳] |
| **حرکت:** | **۱** | **۲** | **۳** |
| **۶۷)** لرزش، مشکل تعادل، مشکل در حرکت و هماهنگی حرکات (لرزش غیرقابل کنترل در بخشی از بدن شما) | نداشتم[۱] | خفیف تا متوسط[۲] | متوسط تا شدید[۳] |
| **خواب:** | **۱** | **۲** | **۳** |
| **۶۸)** مشکلات به خواب رفتن، خواب کوتاهتر یا طولانی تر از حد معمول، وقفه در خواب | هرگز[۱] | گاهی اوقات[۲] | غالب اوقات[۳] |
| **گوش، حلق و بینی:** | **۱** | **۲** | **۳** |

## Persian Version of Data Collection Form- For Cases

| | | | |
|---|---|---|---|
| **۶۹)** گوش درد، وزوز گوش، حساسیت غیر عادی به صداها، کم شنوایی که اخیرا ایجاد شده باشد | نداشتم[۱] | خفیف تا متوسط[۲] | متوسط تا شدید[۳] |
| **۷۰)** تغییر در حس بویایی یا چشایی، عطسه، آبریزش بینی، احتقان در سینوس ها (ناراحتی یا احساس «پری» در اطراف بینی، گونه ها، پیشانی یا اطراف چشم) | نداشتم[۱] | خفیف تا متوسط[۲] | متوسط تا شدید[۳] |
| **۷۱)** تولید خلط ، سرفه، گلودرد، صدای خشن، اشکال در بلع، زخم های دهان، خشکی دهان، بدتر شدن مشکلات دندانی | نداشتم[۱] | خفیف تا متوسط[۲] | متوسط تا شدید[۳] |
| **معده و گوارش:** | **۱** | **۲** | **۳** |
| **۷۲)** شکم درد یا نفخ، حالت تهوع، سوء هاضمه، سوزش سر دل، اسهال، یبوست | نداشتم[۱] | خفیف تا متوسط[۲] | متوسط تا شدید[۳] |
| **۷۳)** کاهش یا افزایش وزن بدون برنامه ریزی | هرگز[۱] | گاهی اوقات[۲] | غالب اوقات[۳] |
| **ماهیچه ها و مفاصل:** | ۱ | ۲ | ۳ |
| **۷۴)** درد یا ضعف یا سفتی عضلات، انقباض یا گرفتگی عضلات، گزگز و بی حسی | نداشتم[۱] | خفیف تا متوسط[۲] | متوسط تا شدید[۳] |
| **۷۵)** درد یا تورم یا سفتی مفاصل | نداشتم[۱] | خفیف تا متوسط[۲] | متوسط تا شدید[۳] |
| **سلامت روان و رفاه:** | **۱** | **۲** | **۳** |
| **۷۶)** لذت نبردن ، غمگینی، نوسانات خلقی، احساس تنهایی، تغییر در اشتها، ناامیدی نسبت به آینده، اضطراب | نداشتم[۱] | خفیف تا متوسط[۲] | متوسط تا شدید[۳] |
| **۷۷)** داشتن افکاری در مورد آسیب رساندن به خود یا احساس تغییر شخصیت نسبت به قبل از ابتلا به کرونا | هرگز[۱] | گاهی اوقات[۲] | غالب اوقات[۳] |
| **پوست و مو:** | **۱** | **۲** | **۳** |
| **۷۸)** خشکی یا خارش پوست | نداشتم[۱] | خفیف تا متوسط[۲] | متوسط تا شدید[۳] |
| **۷۹)** لکه های بنفش قرمز روی پاها، ضایعات پوستی، کهیر | هرگز[۱] | گاهی اوقات[۲] | غالب اوقات[۳] |
| **۸۰)** ریزش مو، تغییر در ناخن ها (ایجاد خطوط عمودی روی ناخن ، ایجاد حفره روی ناخن، تغییر رنگ یا شکنندگی ناخن ها) | نداشتم[۱] | خفیف تا متوسط[۲] | متوسط تا شدید[۳] |
| **چشم:** | **۱** | **۲** | **۳** |

# Persian Version of Data Collection Form- For Cases

| | | | |
|---|---|---|---|
| **۸۱)** قرمزی یا خشکی یا خارش یا آبریزش چشم، فشار پشت چشم، دیدن نور چشمک زن، احساس جسم خارجی در چشم | هرگز[۱] | گاهی اوقات[۲] | غالب اوقات[۳] |
| **۸۲)** درد پشت چشم، تاری دید، دوبینی، حساسیت به نور | نداشتم[۱] | خفیف تا متوسط[۲] | متوسط تا شدید[۳] |
| **سلامت باروری و جنسی:** | **۱** | **۲** | **۳** |
| **زن یا مرد:**<br>**۸۳) در پاسخ دهنده ی زن:** تغییرات در دوره قاعدگی، بدتر شدن علایم پیش از قاعدگی، لخته های خونی بیش از حد معمول در دوران قاعدگی<br>**در پاسخ دهنده ی مرد:** کاهش علاقه به رابطه جنسی، مشکل در انزال، نگرانی در مورد توانایی ارضای جنسی | هرگز[۱] | گاهی اوقات[۲] | غالب اوقات[۳] |
| **زن یا مرد:**<br>**۸۴) در پاسخ دهنده ی زن:** خشکی واژن، ترشح، کاهش علاقه به رابطه جنسی<br>**در پاسخ دهنده ی مرد:** حفظ نعوظ، کاهش علاقه به رابطه جنسی | نداشتم[۱] | خفیف تا متوسط[۲] | متوسط تا شدید[۳] |
| **علائم ادراری:** | **۱** | **۲** | **۳** |
| **۸۵)** از دست دادن کنترل ادرار (نشت)، مشکل در دفع ادرار، دفع ادرار بیشتر از حد معمول | هرگز[۱] | گاهی اوقات[۲] | غالب اوقات[۳] |
| **علائم مربوط به سیستم ایمنی:** | **۱** | **۲** | **۳** |
| **۸۶)** آلرژی شدید (از قبل یا جدید) | هرگز[۱] | گاهی اوقات[۲] | غالب اوقات[۳] |
| **سایر موارد:** | **۱** | **۲** | **۳** |
| **۸۷)** تب یا لرز | هرگز[۱] | گاهی اوقات[۲] | غالب اوقات[۳] |
| **۸۸)** زیاد عرق کردن، گرگرفتگی، تورم غدد لنفاوی، سرگیجه (زمانی که همه چیز در اطراف شما بچرخد به نحوی که تعادل شما را تحت تاثیر قرار دهد) | نداشتم[۱] | خفیف تا متوسط[۲] | متوسط تا شدید[۳] |
| **تأثیر بر زندگی روزانه:** | **۱** | **۲** | **۳** |
| **۸۹)** تغییر در توانایی شما برای انجام کار، رفتن به مدرسه، خرید، انجام کارهای خانه یا کارهای سبک، حرکات آسان، مراقبت از خود، روابط با دوستان و خانواده، لذت بردن از زندگی | اصلا[۱] | تا حدودی[۲] | قابل توجه[۳] |

**۹۰) در حال حاضر، من فکر می کنم که پس از ابتلا به کرونا، به شرایط سلامت قبل از ابتلا به این بیماری برگشته ام.**

۱) اصلاً ۲) خیلی کم ۳) تا حدودی ۴) کاملا

**۹۱) نام و نام خانوادگی پرسشگر .......**

**Persian Version of Data Collection Form- For Controls**

## گروه بدون سابقه ابتلا به کووید-۱۹

**مقایسه شیوع علائم کووید-۱۹ طولانی در بین جمعیت بزرگسال با و بدون سابقه ابتلا به کووید-۱۹ در استان فارس، ۱۳۹۸-۱۴۰۲**

**اطلاعات شخصی:**

**۱) سن:** ....... / **۲) جنسیت:** مرد□[۱] زن□[۲] / **۳) وزن (کیلوگرم) :** ....... / **۴) قد (سانتی متر) :** .......

**۵) سطح تحصیلات:** بی سواد □[۱] کمتر از ششم □[۲] ششم تا یازدهم □[۳] دیپلم دبیرستان □[۴] فوق دیپلم□[۵] لیسانس□[۶] فوق لیسانس □[۷] مدرک دکترا □[۸]

**۶) وضعیت اشتغال:** شاغل□[۱] بیکار□[۲] خانه دار□[۳] دانشجو□[۴] بازنشسته□[۵]

**۷) وضعیت تاهل:** مجرد□[۱] متاهل□[۲] مطلقه□[۳] بیوه□[۴]

**کدامیک از موارد زیر قبل از همه گیری کرونا (یا قبل از ابتلا به کرونا) در مورد شما وجود داشته است؟ (همه موارد را پاسخ دهید.)**

| | ۱ | ۲ |
|---|---|---|
| **۸)** آسم یا سایر بیماری های ریوی | بله[۱] | خیر[۲] |
| **۹)** دیابت | بله[۱] | خیر[۲] |
| **۱۰)** بیماری های قلب و عروق (مانند نارسایی قبل، بیماری های کلیوی، فشار خون بالا) | بله[۱] | خیر[۲] |
| **۱۱)** بیماری های خود ایمنی (مانند لوپوس، روماتیسم) | بله[۱] | خیر[۲] |
| **۱۲)** سرطان (هر نوع) | بله[۱] | خیر[۲] |
| **۱۳)** مصرف دارو (کورتون ها مانند پردنیزولون، داروهای شیمی درمانی) | بله[۱] | خیر[۲] |

**۱۴) وضعیت سیگار کشیدن شما چگونه است؟**

۱) غیر سیگاری (مساوی یا کمتر از ۱۰۰ نخ سیگار در طول عمر)

۲) سیگاری سابق (بیشتر از ۱۰۰ نخ سیگار در طول عمر اما ترک قبل از شیوع کرونا)

۳) سیگاری سابق (بیشتر از ۱۰۰ نخ سیگار در طول عمر اما ترک پس از شیوع کرونا و حداقل یک ماه است که سیگار نمیکشم )

۴) سیگاری فعلی (بیشتر از ۱۰۰ نخ سیگار در طول عمر و مصرف کننده فعلی)

**************************** هنگام ورود داده ها سه شماره خالی نگه داشته شود**********************************

**سابقه پزشکی شما**

**۱۸) وضعیت سلامت شما به طور کلی تا ۳ ماه قبل از شیوع کرونا در اسفند ۱۳۹۸، چگونه بود؟**

۱) خیلی خوب ۲) خوب ۳) متوسط ۴) ضعیف

**در طول دوره پاندمی و تا کنون ، کدام یک از علائم مربوط به سوالات ۱۹ تا ۴۷ را داشتید ؟**

| تنفس : | ۱ | ۲ | ۳ |
|---|---|---|---|
| **۱۹)** تنگی نفس | نداشتم[۱] | خفیف تا متوسط[۲] | متوسط تا شدید[۳] |
| **۲۰)** تنفس سریعتر از حد معمول یا بیدار شدن از خواب به دلیل تنگی نفس | هرگز[۱] | گاهی اوقات[۲] | غالب اوقات[۳] |

# Persian Version of Data Collection Form- For Controls

| | | | |
|---|---|---|---|
| **درد :** | **۱** | **۲** | **۳** |
| **۲۱)** درد قفسه سینه، درد در هنگام تنفس، درد در هر نقطه از بدن، درد در تمام بدن ، سر درد | نداشتم[۱] | خفیف تا متوسط[۲] | متوسط تا شدید[۳] |
| **گردش خون :** | **۱** | **۲** | **۳** |
| **۲۲)** تپش قلب، احساس ضعف، احساس سرگیجه حین ایستادن، ورم اندام ها یا صورت یا لب ها یا زبان یا گلو ، اندام های سرد (که بیشتر از حد معمول طول بکشد یا سرد تر از حد معمول باشد) هیچ خفیف متوسط شدید | نداشتم[۱] | خفیف تا متوسط[۲] | متوسط تا شدید[۳] |
| **خستگی :** | ۱ | ۲ | ۳ |
| **۲۳)** خستگی شدید ، احساس خستگی جسمی یا روحی که با استراحت بهبود نمی یابد ، بدتر شدن علائم شما به دنبال فعالیت های فیزیکی یا ذهنی ساده | نداشتم[۱] | خفیف تا متوسط[۲] | متوسط تا شدید[۳] |
| **حافظه، تفکر و ارتباط:** | **۱** | **۲** | **۳** |
| **۲۴)** مه مغزی (احساس کندی ، خالی شدن فکر)، گیجی، مشکلات حافظه ، مشکل در تمرکز یا برنامه ریزی، مشکلات کلمه یابی حین صحبت کردن | نداشتم[۱] | خفیف تا متوسط[۲] | متوسط تا شدید[۳] |
| **۲۵)** مشکل در درک آنچه دیگران می گویند، گفتار نامفهوم، مشکل در خواندن | نداشتم[۱] | خفیف تا متوسط[۲] | متوسط تا شدید[۳] |
| **حرکت:** | **۱** | **۲** | **۳** |
| **۲۶)** لرزش، مشکل تعادل، مشکل در حرکت و هماهنگی حرکات (لرزش غیرقابل کنترل در بخشی از بدن شما) | نداشتم[۱] | خفیف تا متوسط[۲] | متوسط تا شدید[۳] |
| **خواب:** | **۱** | **۲** | **۳** |
| **۲۷)** مشکلات به خواب رفتن، خواب کوتاهتر یا طولانی تر از حد معمول، وقفه در خواب | هرگز[۱] | گاهی اوقات[۲] | غالب اوقات[۳] |
| **گوش، حلق و بینی:** | **۱** | **۲** | **۳** |
| **۲۸)** گوش درد، وزوز گوش، حساسیت غیر عادی به صداها، کم شنوایی که اخیرا ایجاد شده باشد | نداشتم[۱] | خفیف تا متوسط[۲] | متوسط تا شدید[۳] |
| **۲۹)** تغییر در حس بویایی یا چشایی، عطسه، آبریزش بینی، احتقان در سینوس ها (ناراحتی یا احساس «پری» در اطراف بینی، گونه ها، پیشانی یا اطراف چشم) | نداشتم[۱] | خفیف تا متوسط[۲] | متوسط تا شدید[۳] |
| **۳۰)** تولید خلط ، سرفه، گلودرد، صدای خشن، اشکال در بلع، زخم های دهان، خشکی دهان، بدتر شدن مشکلات دندانی | نداشتم[۱] | خفیف تا متوسط[۲] | متوسط تا شدید[۳] |
| **معده و گوارش:** | **۱** | **۲** | **۳** |
| **۳۱)** شکم درد یا نفخ، حالت تهوع، سوء هاضمه، سوزش سر دل، اسهال، یبوست | نداشتم[۱] | خفیف تا متوسط[۲] | متوسط تا شدید[۳] |

# Persian Version of Data Collection Form- For Controls

| | | | |
|---|---|---|---|
| **۳۲)** کاهش یا افزایش وزن بدون برنامه ریزی | هرگز[۱] | گاهی اوقات[۲] | غالب اوقات[۳] |
| **ماهیچه ها و مفاصل:** | ۱ | ۲ | ۳ |
| **۳۳)** درد یا ضعف یا سفتی عضلات، انقباض یا گرفتگی عضلات، گزگز و بی حسی | نداشتم[۱] | خفیف تا متوسط[۲] | متوسط تا شدید[۳] |
| **۳۴)** درد یا تورم یا سفتی مفاصل | نداشتم[۱] | خفیف تا متوسط[۲] | متوسط تا شدید[۳] |
| **سلامت روان و رفاه:** | **۱** | **۲** | **۳** |
| **۳۵)** لذت نبردن ، غمگینی، نوسانات خلقی، احساس تنهایی، تغییر در اشتها، ناامیدی نسبت به آینده، اضطراب | نداشتم[۱] | خفیف تا متوسط[۲] | متوسط تا شدید[۳] |
| **۳۶)** داشتن افکاری در مورد آسیب رساندن به خود یا احساس تغییر شخصیت نسبت به قبل از ابتلا به کرونا | هرگز[۱] | به ندرت[۲] | گاهی[۳] |
| **پوست و مو:** | **۱** | **۲** | **۳** |
| **۳۷)** خشکی یا خارش پوست | نداشتم[۱] | خفیف تا متوسط[۲] | متوسط تا شدید[۳] |
| **۳۸)** لکه های بنفش قرمز روی پاها، ضایعات پوستی، کهیر | هرگز[۱] | گاهی اوقات[۲] | غالب اوقات[۳] |
| **۳۹)** ریزش مو، تغییر در ناخن ها (ایجاد خطوط عمودی روی ناخن ، ایجاد حفره روی ناخن، تغییر رنگ یا شکنندگی ناخن ها) | نداشتم[۱] | خفیف تا متوسط[۲] | متوسط تا شدید[۳] |
| **چشم:** | **۱** | **۲** | **۳** |
| **۴۰)** قرمزی یا خشکی یا خارش یا آبریزش چشم، فشار پشت چشم، دیدن نور چشمک زن، احساس جسم خارجی در چشم | هرگز[۱] | گاهی اوقات[۲] | غالب اوقات[۳] |
| **۴۱)** درد پشت چشم، تاری دید، دوبینی، حساسیت به نور | نداشتم[۱] | خفیف تا متوسط[۲] | متوسط تا شدید[۳] |
| **سلامت باروری و جنسی:** | **۱** | **۲** | **۳** |
| **زن یا مرد:**<br>**۴۲) در پاسخ دهنده ی زن:** تغییرات در دوره قاعدگی، بدتر شدن علایم پیش از قاعدگی، لخته های خونی بیش از حد معمول در دوران قاعدگی<br>**در پاسخ دهنده ی مرد:** کاهش علاقه به رابطه جنسی، مشکل در انزال، نگرانی در مورد توانایی ارضای جنسی | هرگز[۱] | گاهی اوقات[۲] | غالب اوقات[۳] |
| **زن یا مرد:**<br>**۴۳) در پاسخ دهنده ی زن:** خشکی واژن، ترشح، کاهش علاقه به رابطه جنسی | نداشتم[۱] | خفیف تا متوسط[۲] | متوسط تا شدید[۳] |

# Persian Version of Data Collection Form- For Controls

| | | | |
|---|---|---|---|
| **در پاسخ دهنده ی مرد:** حفظ نعوظ، کاهش علاقه به رابطه جنسی | | | |
| **علائم ادراری:** | **۱** | **۲** | **۳** |
| **۴۴)** از دست دادن کنترل ادرار (نشت)، مشکل در دفع ادرار، دفع ادرار بیشتر از حد معمول | هرگز[1] | گاهی اوقات[2] | غالب اوقات[3] |
| **علائم مربوط به سیستم ایمنی:** | **۱** | **۲** | **۳** |
| **۴۵)** آلرژی شدید (از قبل یا جدید) | هرگز[1] | گاهی اوقات[2] | غالب اوقات[3] |
| **سایر موارد:** | **۱** | **۲** | **۳** |
| **۴۶)** تب یا لرز | هرگز[1] | گاهی اوقات[2] | غالب اوقات[3] |
| **۴۷)** زیاد عرق کردن، گرگرفتگی، تورم غدد لنفاوی، سرگیجه (زمانی که همه چیز در اطراف شما بچرخد به نحوی که تعادل شما را تحت تاثیر قرار دهد) | نداشتم[1] | خفیف تا متوسط[2] | متوسط تا شدید[3] |

*****************************هنگام ورود داده ها هشت شماره خالی نگه داشته شود*************************************

**واکسیناسیون**

**۵۶) آیا واکسن کرونا را تزریق کرده اید؟**

۱) خیر، من هیچ دوزی دریافت نکردم ۲) بله، فقط یک دوز ۳) بله، دو دوز ۴) بله، سه دوز یا بیشتر

**۵۷) در چه تاریخی واکسن کرونا را تزریق کردید ؟ اگر دقیقاً به خاطر نمی آورید، لطفاً بهترین تخمین خود را ارائه دهید. (میتوانید بیش از یک مورد را علامت بزنید).**

۱) بار اول: -- / -- / ---- ۲) بار دوم: -- / -- / ----

۳) بار سوم: -- / -- / ---- ۴) بار چهارم: -- / -- / ----

*****************************هنگام ورود داده ها سی و دو شماره خالی نگه داشته شود ************************************

**۹۰) در حال حاضر، من فکر می کنم که وضعیت سلامت من مانند دوران قبل از شیوع کرونا در اسفند ۱۳۹۸ می باشد.**

۱) اصلاً ۲) خیلی کم ۳) تا حدودی ۴) کاملا

**۹۱) نام و نام خانوادگی پرسشگر .....**
